# Fern rhizomes as fodder in Norway

**DOI:** 10.1186/s13002-016-0112-0

**Published:** 2016-09-06

**Authors:** Torbjørn Alm

**Affiliations:** Tromsø museum, University of Tromsø, PO Box 6050, Langnes, N-9037 Tromsø, Norway

**Keywords:** Pteridophytes, Rhizomes, Fodder, *Dryopteris expansa*, *Dryopteris filix-max*, *Matteuccia struthiopteris*, *Athyrium filix-femina*

## Abstract

**Background:**

Although ferns are often known under collective names in Norway, e.g. *blom*, a substantial number of vernacular names for individual fern species are known, in particular for useful or poisonous taxa. In the past, the rhizomes (Norwegian: *moldfôr*) of selected species were collected for fodder. Only scattered records of such use are available from southern Norway, and the tradition’s core area is found in the two North Norwegian counties of Nordland and Troms, in accordance with the longer winters encountered in the north, frequently leading to fodder shortage in early spring. The tradition extends northeastwards into Finnmark, but is less well documented there. Although numerous sources mention the use of fern rhizomes for fodder, the fern species hiding behind the tradition are incompletely known. This paper aims at reviewing available data in terms of identifyng the species used for fodder, the history and geographical distribution of such use, and other relevant traditions, e.g. the timing and mode of collection, and the way the rhizomes were used.

**Methods:**

The study is based on data extracted from a variety of archival and literature sources; the latter retrived from my database of more than 7500 publications providing information on plant names and plant uses in Norway.

**Results:**

More than 200 individual records mention the use of fern rhizomes for fodder in Norway. Only a fraction of these, typically made by botanist recording data on plant uses, provides information on the identity of the species used. Based on these, *Dryopteris filix-mas* and *Matteuccia struthiopteris* stand out as the most important species serving as sources of fern rhizomes for fodder. Locally, *Dryopteris expansa* was the preferred species, and this taxon may to some extent be overlooked in the records so far available. With a few exceptions, Norwegian folk tradition singles out *Athyrium filix-femina* as a harmful and poisonous species, causing livestock to go blind and lame, but whether this is true or not, remains unknown; the symptoms are in fact documented elsewhere as a consequence of poisoning due to *Dryopteris filix-mas*. In coastal north Norway, fern rhizomes were regularly collected for fodder, both in late autumn and early spring, and used to remedy a recurrent shortage of fodder in late winter and spring. Locally, the tradition of collecting fern rhizomes lived on until the 1940’s or 1950’s. Although mainly a tradition of the ethnic Norwegians, it had also been adopted by the farmers belonging to the Finnish and Sámi ethnic minorities.

**Conclusion:**

Fern rhizomes have a long tradition as an additional fodder for livestock in Norway. Preferred species were *Matteuccia struthiopteris* and *Dryopteris filix-mas*, locally also *Dryopteris expansa. Athyrium filix-femina* was considered to be poisonous, and usually avoided.

## Background

Ferns form an important part of the flora and vegetation of Norway, in particular in the humid coastal areas. *Athyrium filix-femina* (L.) Roth, *Dryopteris expansa* (C. Presl.) Fraser-Jenk. & Jermy, *Matteuccia struthiopteris* (L.) Tod., and other taxa may predominate in luxuriant forest vegetation and on the lower mountain slopes. Conspicuous and abundantly available, such species have been well known to farmers during past times of subsidence economy, which in coastal Norway was largely based on a combination of agriculture and fisheries.

Ferns are usually avoided by grazing animals, and the fronds were not generally scythed or otherwise collected for fodder. Nonetheless, fern species formed an important supplementary source of livestock fodder in Norway, in particular in the north. Rhizomes of several species were dug up and boiled, usually together with other foodstuffs of the most diverse kind, ranging from fish remains and kelp to twigs and bark of various deciduous trees, heather and seaweeds.

This paper is a review of ethnobotanical data on the uses of pteridophyte or fern rhizomes for fodder in Norway. Altogether, 53 species and subspecies of ferns are known from Norway [1]. Ethnobotanical traditions are related only to a fraction of these, mainly species of Aspleniaceae (*Asplenium* spp.), Blechnaceae (*Blechnum spicant* (L.) Roth), Dennstaedtiaceae (*Pteridium aquilinum* (L.) Kuhn), Dryopteridaceae (*Dryopteris* spp., especially *D. filix-mas* (L.) Schott), Polypodiaceae (*Polypodium vulgare* L.), and Woodsiaceae (*Athyrium filix-femina* and *Matteuccia struthiopteris*). The tiny species of Ophioglossaceae (*Botrychium* spp., *Ophioglossum vulgatum* L.) form a separate group, and have been excluded here. Norwegian traditions related to *Botrychium* spp. are discussed by Rolf Nordhagen [2]. There are no traditions related to using *Asplenium* spp. or *Polypodium vulgare* as fodder, so these are not relevant in the present context.

## Methods

Only a few ethnobotanical studies provide extensive information on ferns. Jens Holmboe [3] carried out a special study devoted to the use of fern rhizomes, noting that farmers he met during his travels in northern Norway in 1910–11 and 1914–15 were still collecting and using fern rhizomes. This made it possible for him to identify the species used, which is difficult or impossible from literature records. Unfortunately, Holmboe’ brief paper leaves much to be desired. He provides no details in terms of the number of informants the study is based on, referring only to vague quantities like “numerous farmers”, “in some cases”, and little in terms of geographical details, though he seemingly gathered most of his information in the Bardu and Målselv area of interior Troms.

Since then, much new information has been collected. An important source is found in the responses to a questionnaire (No. 11) on various additional fodders, distributed by Norsk etnologisk gransking (Norwegian ethnological survey) in 1948 (referred to here as NEG 11 + record number). The three-page questionnaire includes a separate section on ferns as fodder. It was distributed to a substantial network of informants, and yielded more than 200 answers from all parts of Norway. Although some replies cover only specific topics of interest to the informant, leaving the rest blank, most tried to answer all questions, thus providing an important source in terms of where fern rhizomes as fodder were still remembered in the mid-20th century,

The botanist Ove Arbo Høeg’s vast collection of ethnobotanical data, mainly from the 1940’s, is another important source. His original material is deposited in Norsk folkeminnesamling (Norwegian folklore collection), and referred to here as NFS O.A. Høeg and record number; an extensive summary of the material was published in 1974 [4]. The citations in Høeg’s compilation are often considerably edited and altered versions of those found in the original material, and he does not indicate the source or record/informant number. I have frequently preferred to cite the source material, referring to record numbers and the year the record was made (e.g. NFS O.A. Høeg 485; 1938). Høeg also included some data from the NEG material, but much was left out, and has never been published or utilized.

I have added data from my own extensive field work and correspondence, and the resulting collection of ethnobotanical material from Norway, of which only a tiny fraction has been published (e.g. [5, 6]) or used in publications on various species and topics (e.g. [7–14]). These records are referred to by the acronym EBATA, followed by year and record number (e.g. EBATA 1990:9). I have also incorporated data from material collected by Brynhild Mørkved in the early 1990’s (EBABM series). Both data sets are housed at Tromsø museum, University of Tromsø. Furthernmore, I have extracted data from the more than 7500 references presently incorporated in my database of literature providing information on plant names and uses in Norway.

As far as possible, I have gone through all archival and literature sources known to me, extracting information related to ferns. It is not possible for any single person to scour the entire national literature of a single country, but by now, my reading of Norwegian sources is very extensive, and tens of thousands of literature excerpts relating to plants and plant uses have been entered into a vast database. For the purpose of this paper, I have included every single piece of information related to fern rhizomes, faithfully including the few records which deviate from the general pattern in terms of the species used. As discussed in the section on vernacular names, there is an inherent problem in the material as such, since records (and place-names) related to *moldfôr* (‘soil fodder’) almost by definition refer to fern rhizomes and their use, whereas a number of other Norwegian (and Finnish orf Sámi) fern terms may refer both to fern rhizomes and the above-ground parts. Thus, data on the latter group need to include additional information to reveal if the record refers to some kind of fodder use of fern rhizomes. The problem of identifying the species is dealt with extensively below; in general, only informants or recorders with some kind of botanical training have ventured to name the species hiding behind the vernacular names. The material has been arranged and analysed accordingly, allowing the multitude of records related to unidentified species to provide a variety of information on the use of fern rhizomes, e.g. on tools needed, time of harvest, moods of use etc., and providing a much better picture of the general distribution of the tradition than the “identified” records alone would allow. As far as possible, all records are referred to counties (for a map, see Fig. 6) and municipalities.

## Results

### Vernacular names

In Norwegian folk tradition, ferns (except *Botrychium*) are often merged into a single ethnotaxonomical unit. In general, when using such terms, people have been surprisingly successful in circumscribing the otherwise rather variable representatives of the various fern families that were previously included in Polypodiaceae s.l. (see e.g. [5]: 378). In Western and North Norway, *blom* is a widely used, collective term for all kinds of ferns ([4]: 323ff, ([15]: 32, [16]: 86, [17]: 3, [18]: 138). It is prevalent along the entire western coast of Norway, extending eastwards to the coastal areas of E Finnmark in northernmost Norway. Locally, the term may also have been used in interior south Norway, as suggested e.g. by Asbjørn Hagen ([19]: 219) and some toponyms ([20]: 222). Sometimes, the smaller species were termed *småblom*, i.e. “small fern”, e.g. at Sunnmøre in Western Norway ([19]: 219). Other, related terms may also occur, e.g. *fugleblom* (“bird bloom”) in Dalsfjord (Volda), Møre og Romsdal ([19]: 219) and *kalvablom* (“calf bloom”) for *Dryopteris filix-mas* in Os, Hordaland ([19]: 219).

Apart from *blom*, a number of other collective names for ferns exist ([21]: 408–409). These include *gjeiske*, which is widely dispersed in Norway, in various local versions ([3]: 764, [4]: 332), including *gjiske* ([19]: 220), and *gjeske* in Nordland ([15]: 32), [22]: 81), e.g. as *Kaal-jæske* at Helgeland, or *kålgjeske* (‘cabbage fern’) in modern Norwegian ([23]: 288). Another widely distributed term is *ormegras* or ‘worm grass’, also in various dialectal versions, mainly in SE Norway. Variants of the latter include *ormgras* ([19]: 220, [24]: 4) and *ørmegras* in Valdres, E Norway ([19]: 219). Deviations occur, e.g. at Fitjar in Hordaland, where most people used *einstabbe* or *einstape* – otherwise a widespread name for *Pteridium aquilinum* – as a common term for all larger ferns (EBATA 2006:41).

Ferns are generally under-specified in Norwegian folk taxonomy. Only a few useful or otherwise noteworthy species were recognized and given separate names, and thus identified at a one-to-one level. Fronds were generally of little interest, and only locally scythed for fodder. The rhizomes, however, have been frequently collected, and formerly constituted an important source of additional fodder, in particular in the northern parts of Norway. Useful fern rhizomes are generally known as *mollfôr* or *moldfôr. Mollfôr* is the prevailing spelling, although *moldfôr* would be more in accordance with standard Norwegian, i.e. ‘soil fodder’. I have retained deviant spellings in citations, so a variety of forms will be encountered below. The name, in its Norse form *moldfoðr*, can be traced back to medieval times. The first written record occurs in a document from 1293; see below. The term is widely distributed in northern Norway, within the main area of the tradition related to rhizomes as fodder ([4]: 333, [25]: 26).

*Telg* (also *tilg*, *tælg*, *kjelg* or *kjælg*) is another widely distributed vernacular name for ferns and/or fern rhizomes in Norway ([4]: 331, ([15]: 32, [24]: 4). Sometimes, it is the only name in use, e.g. *tælg* in Valdres, E Norway ([19]: 219), but it may also occur as an alternative, second name in areas where *moldfôr* predominates (e.g. [5]: 378). According to Göran Wahlenberg, *tilg*, *tælg* and *molfoor-tælg (= moldfôrtelg)* were used as names for *Matteuccia struthiopteris* in Troms ([23]: 288). Other general terms for ferns include *grofte* in central Norway and Nordland ([4]: 332), and *kjag* in southeast Norway ([4]: 331).

Within North Norway, the term *moldfôr* is widespread and well known at least from Northern Nordland through Troms into western Finnmark ([3]: 764, [4, 26], and other sources cited below). Place-names comprising *moldfôr* (see also below) indicate that the term at least formerly was known in southern Nordland as well ([3]: 764, [27]: 54).

*Burkn*, which is mainly used in southernmost and western Norway ([4]: 331, [15]: 32), in many dialectal variations, may also reflect the use of fern rhizomes. The name is related to the verb *burka*, “chop” ([28]: 107) – and the only part of ferns that needs chopping (see below) is the rhizome. Both *burkn* and *burknrót* are known from the Norse medicinal literature ([29]: 8, 96), referring to *Dryopteris filix-mas. Burkn* is also found in several toponyms in the southern part of Norway ([30]: 235).

With eroding knowledge of the old practice of collecting *moldfôr* or fern rhizomes for fodder, people are increasingly unfamiliar with the term as such, and its rather obvious meaning. Thus, deviant forms have developed, e.g. *moltfôr* ([31]: 77), a spelling that has now also started appearing on maps, even at sites where the *mold-* spelling is easily documented as the old and traditional one.

Several informants provide descriptions of the kind of fern rhizomes used, but these are often difficult to “translate” into species. An example from Lierne (Nord-Trøndelag) may be quoted: “I am not aware that there is more than one kind. It has root which become as large as a medium-sized rutabaga. But the roots are in layers outside each other. Their outside is black, but inside they are pale green. It is called *grøftrot*.” (NFS O.A. Høeg 375; 1940). From Sortland in Nordland, Ingvald Johansen provides a rather similar description of the kind of fern rhizome that was collected: “But *moldfôret* is the root beneath the *bregne* [fern], or the *blom* as it was also called. The size differs according to the age of the plant, but it may be 15 to 20 cm long, and looks a little like an ananas. Outside, it is dark brown, but if it is chopped into pieces, the interior has a pale green colour. The taste is bitter, but it was much used as an additional fodder on the farms in the [Holmstaddalen] valley” ([32]: 100).

Locally, people would discriminate between two or more kinds of fern rhizomes, and most would know that only certain fern species could or should be used. Unfortunately, vouchers specimens and detailed records that allow the species to be identified are few and far between. A review of this aspect is a primary goal of this paper, adding details on how and when the collection of rhizomes was done, the way they were used, and the geographical distribution of the practice.

The Sámi and Finnish etnhnic minorities also have terms for fern rhizomes. The North Sámi *gáiski* and Finnish *kaiski* are closely related, and the most frequent terms. As noted for Sámi by Just Qvigstad, the term is a Norse loan-word ([33]: 159). He noted *reppe* (in present-day spelling: *rehppe*) as an additional Sámi term for fern rhizomes in Hamarøy, Nordland ([34]: 318, 320) and Skånland, Troms ([35]: 125), and *delgi* e.g. in the Ofoten area of Nordland and in Skånland, Troms ([34]: 309, [35]: 125); the latter again obviously a Norse loan-word (from *telg*).

### *Moldfôr* in toponyms

Unsurprisingly, since fern rhizomes were considered a valuable source of additional fodder, areas with abundant fern resources might be named accordingly (cf. [4]: 329). Toponyms comprising the term *moldfôr* are found at numerous locations in northern Norway (Table 1), though perhaps not in hundreds of sites, as suggested by Ottar Brox ([36]: 8). Examples are found at Brekke in Skjerstad (Bodø), Nordland, “where there is a place called Moldforbakkan, which suggests that the roots have been used” (EBATA 2010:40), at Mollforskaret in Sørfold, Nordland ([37]: 44), and Moldfôrlia at Alvestad, Grytøya in Harstad, Troms [38]. In Gratangen, Troms, a similar locality was used as a source or rhizomes: “There you will also find Mollforholla. They dug roots there, for fodder.” (EBATA 2005:9). A boulder at Reinøya in Karlsøy was called Moldfôrsteinen (EBATA 2016:6). At Arnøya in Skjervøy, Troms, a large bay is called Moldforvika, but people in an adjacent area had no idea what the name referred to (EBATA 2005:44).

**Table 1:** Norwegian toponyms referring to fern rhizomes (*moldfôr*)

Toponym	Location	Map sheet & coordinates (MGRS grid reference) if available	Literature/source
^*^Moldfôrbakken	Troms: Balsfjord: Josefvannet	1533 II, 69°17’ N, 19°30’ E (DB 30,86)	
^*^Moldfôrbakken	Troms: Lyngen: Sørlenangen	1634 IV, 69°46’ N, 19°20’ E (DC 61,40)	
Moldfôrbakken	Troms: Kåfjord: Manndalen		[27]: 54
Moldfôrbakken	Alta: Talvik: Tappeluft		[27]: 54
Moldfôrbakkvatnet	Nordland: Hadsel: Hinnøya: Raftsundet	1231 IV, 68°25’ N, 14°11’ E (WR 07,89)	
Moldfôrbekken	Troms: Salangen: Nervatnet	1432 IV, 68°53’ N, 17°54’ E (XS 16,43)	[27]: 54
Moldfôrbukta	Nordland: Beiarn: Moldjord	2029 III, 67°04’ N, 14°36’ E (VQ 82,38)	
^*^Moldfôrdalen	Troms: Salangen: Nervatnet	1432 IV, 68°53’ N, 17°54’ E (XS 16,44)	
Moldfôrdalen	Finnmark: Alta: Bossekop	-	[27]: 54, [39]: 18
Moldfôrelva	Finnmark: Alta	-	[27]: 54, [39]: 40
Moldfôrhaugen	Troms: Salangen: Salangsdalen	-	[27]: 54, [40]: 56
Moldfôrhellaren	Troms: Tromsø: Oldervik	-	[27]: 54
Moldfôrholla	Troms: Skånland	-	NFS O.A. Høeg 785
Moldfôrholla	Troms: Gratangen		EBATA 2005:9
indre Moldforholmen	Nordland: Bodø: Karlsøyvær	2030 III, 67°34 N, 14°40’ E (VQ 85,94)	
ytre Moldforholmen	Nordland: Bodø: Karlsøyvær	2030 III, 67°34 N, 14°39’ E (VQ 85,95)	
^*^Moldfôrhågen	Nordland: Narvik: Håkvikdalen	1331 I, 68°23’ N, 17 ^o^20 E (WR 9568,872)	
^*^Moldfôrhågen	Nordland: Narvik: Håkvikdalen	1331 I, 68°24’ N, 17 ^o^22 E (WR 968,890)	
^*^Moldfôrhågen	Troms: Salangen: Røyrbakkvannet	1432 IV, 68°58’ N, 17°45’ E (XS 098,550)	
Moldfôrkløft	Finnmark: Alta: Aronnes	-	[27]: 54
Moldfôrland	Nordland: Steigen: Vinkfjorden	2130 III, 67°16’ N, 18°48’ E (DB 130,850)	[27]: 54
^*^Moldfôrlia	Troms: Balsfjord: Aursfjorden	1533 IV, 69°55 N, 20 ^o^ 51’ E (DC 94,56)	
Moldfôrnes	Finnmark: Alta: Leirbotn	1935 III, 70°07’ N, 23°26’ E (EC 92,80)	
^*^Moldfôrnes[et]	Nordland: Beiarn: Beiarndalen	2028 IV, 66°51’ N, 14°40 E (VQ 85,14)	
[27]: 54			
Moldfôrneset	Troms: Skjervøy	-	[27]: 54
Moldfôrneset	Troms: Nordreisa: Maurneset	1634 I, 69°55’ N, 20 ^o^ 51’ E (DC 94,56)	
^*^Moldfôrneshaugen	Finnmark: Alta: Leirbotn	1935 III, 70°07’ N, 23°26’ E (EC 92,80)	-
^*^Moldfôrnesholmen	Finnmark: Alta: Leirbukt	-	[27]: 54
Moldfôrskaret	Nordland: Sørfold: Sagfjorden	2130 III, 67°34’ N, 15 ^o^26’ E (WQ 18,94)	
^*^Moldfôrsletten	Troms: Dyrøy: Dyrøysundet	1433 III, 69°04’ N, 17 ^o^38’ E (XS 04,62)	[27]: 54, [40]: 58
^*^Moldfôrslåtta	Finnmark: Alta: Transfardalen		[27]: 54, [39]: 40
^*^Moldfôrsteinen	Sortland:	-	[32]: 100
Moldfôrvika	Nordland: Beiarn: Beiarfjorden	2029 III, 67°04’ N, 14 ^o^36 E (VQ 82,38)	
^*^Moldfôrvika	Nordland: Narvik: Skjomen	1331 I, 68°22’ N, 17 ^o^16 E (WR 91,85)	-
^*^Moldfôrvika	Troms: Salangen: Sagfjorden	1432 IV, 68°52’ N, 17 ^o^42 E (XS 09,42)	-
Moldfôrvika	Troms: Nordreisa: Maursundet	1634 I, 69°55’ N, 20 ^o^ 50’ E (DC 93,54)	-
^*^Moldfôrvika	Troms: Kvænangen: Bankenes	1734 I, 69°54’ N, 21 ^o^ 53’ E (EC 34,55)	[27]: 54
lille Moldfôrvika	Troms: Skjervøy: Arnøya	1635 II, 70°13’ N, 20 ^o^ 47’ E (DC 91,89)	cf. [27]: 54
store Moldfôrvika	Troms: Skjervøy: Arnøya	1635 II, 70°13’ N, 20 ^o^ 46’ E (DC 91,90)	
^*^Moldfôrvikdalen	Nordland: Ballangen: Efjorden	1331 IV, 68°18’ N, 16°18 E (WR 538,782)	
Moldfôrvikelva	Troms: Salangen: Sagfjorden	1432 IV, 68°52’ N, 17°42’ E (XS 08,42)	
^*^Moldfôrvikneset	Troms: Salangen: Sagfjorden	1432 IV, 68°52’ N, 17 ^o^42 E (XS 09,42)	
^*^Moldfôrvikodden	Nordland: Beiarn: Beiarfjorden	2029 III, 67°04’ N, 14 ^o^36 E (VQ 82,38)	
Moldførneset	Nordland: Bodø: Eidevågen	2030 II, 67°34 N, 15° 0’ E (VQ 99,94)	
^*^Moltfôrbakken	Nordland: Narvik: Herjangen	1431 IV, 68°30’ N, 17 ^o^32 E (XS 039,001)	
^*^Moltforrbakkhøgda	Nordland: Narvik: Herjangen	1431 IV, 68°30’ N, 17 ^o^32 E (XR 038,998)	
^*^Moltforrbakkskaret	Nordland: Narvik: Herjangen	1431 IV, 68°30’ N, 17 ^o^32 E (XR 038,999)	
^*^Moltforrelva	Nordland: Ballangen	-	[31]: 77
^*^Moltforrhøgda	Nordland: Ballangen: Saltvannet	1331 I, 68°22’ N, 16 ^o^50 E (WR 760,847)	
^*^Moltfôrsteinen	Nordland: Bodø: Kjerringøy	-	NFS O.A. Høeg 571
Moltfôrvik	Nordland: Ballangen: Ofotfjorden	1331 IV, 68°26’ N, 16°30’ E (WR 50–61,89)	[31]: 77
Moldfôråsen	Troms: Skånland	1332 III, 68°33 N, 16°44’ E (WS 70,04)	NFS O.A. Høeg 785; [25]: 26

Names marked * are not shown in the standard N50 map series, but included on more detailed maps, or mentioned in literature or archival sources

In his survey of toponyms in northern Norway comprising the names of plant or animals, Just Qvigstad ([27]: 54) recorded 17 localities based on *moldfôr*. The name index accompanying the main Norwegian map series (N50) lists 14 toponyms which include the term *moldfôr*. All are in northern Norway, and all are at the coast of the Nordland and Troms counties, mostly situated in the fjords and some of the larger islands, i.e., along the coast. Two outer-coast islets in the Karlsøyvær archipelago of Bodø, Nordland, form an exception in terms of location and topography. Some further names are mentioned in various literature sources (e.g. [39]: 40, [40]: 58). In reality, there are probably many more such toponyms, often referring to minor sites, too small to be included on topographical maps at the 1:50 000 scale; or not yet recorded by the map-makers.

In addition, numerous toponyms containing fern terms like *grofte*, *jeiske* and *telg* may refer to sites where rhizomes were collected – or simply to large stands of ferns, since the terms are not necessarily used only for the for rhizomes, as shown by the frequent use of terms like *jeiskrot* (‘*jeisk* root’), *telgrot* (‘*telg* root’) etc. They cannot, thus, be taken as indicating former use of fern rhizomes, even if this is the most likely origin of such toponyms. The same goes for Sámi and Finnish toponyms based on fern terms; they may refer to large stands, or former collecting grounds.

### History

In the distant past, fern rhizomes were probably collected for fodder in large parts of Norway, in particular in the humid coastal areas, where ferns generally occur in abundance. The oldest record of such use anywhere in Norway is related to the Hardanger area of Hordaland in the far southwest, where a document dated 1293 deals with the ownership of a land area, and the right to collect fern rhizomes there. It is included in vol. 4 of *Diplomatarium norvegicum* ([41]: 9). Thus, fern rhizomes must have been of some economic importance more than 700 years ago. From Sunnfjord in Sogn og Fjordane, western Norway, Hans Arentz in 1802 mentioned *tælgroed* (in modern Norwegian: *telgrot*, i.e. ‘fern root’) as the “best means of getting through times of fodder shortage” ([42]: 88). Jens Holmboe ([3]: 763) commented that the term *moldfôr* had since been forgotten in the entire southern part of Norway. This is not entirely correct, although only a few modern records confirm the survival of the name and the old practice (see below). The term is certainly much more widely known in northern Norway.

Bishop Johan Ernst Gunnerus, author of the first Norwegian flora [43, 44], considered *Dryopteris filix-mas* the best source of fern rhizomes for fodder. In a letter to Linnaeus, dated May 19, 1764, he commented on the the plan for the first volume of his projected *Flora norvegica*, and the intention to include figures there. The first two of these should show *Matteuccia struthiopteris* and *Dryopteris filix-mas*; the aim was to ensure that people did not mistake these, and in particular the last one, “the very best *filix*, with *filix femina* [*Athyrium filix-femina*] and other similar and harmful [species]” (cited from [45]: 42). Note, however, that based on the shape and size of the rhizomes, the most likely source of mistaking material is by confusing *Matteuccia struthiopteris* and *Athyrium filix-femina*; both with thick, large and upright rhizomes which may to some extent protrude above ground.

During an excursion to western Norway in 1773, Gerhard Schøning visited a farm where “edible roots” were collected, recorded as *maullfoor* in his extensive travel account ([46]: 95). Although he named the species as *Polypodium vulgare*, his description of the rhizome (“the root or the claws of the root [i.e. the basal part of the stipe] has no bags, when you let your fingers run over it”) suggests that the rhizomes derived from a *Dryopteris* species, assumed to be *D. filix-mas* by Jens Holmboe ([3]: 763).

Further north, most records related to fern rhizomes as fodder are modern, and have been made during the 20th century. A few exceptions occur. In 1743, the authorities in Copenhagen distributed a questionnaire intended to collect data for a comprehensive topographical description of Norway. The replies have recently been published in a five volume-set [47–51]. Whereas some aspects of local agriculture, e.g. the cultivation of barley, rye etc., are well covered, fodders received little attention. Only a small fraction of the many local accounts of parishes and other administrative units provide such details. Peter Schnitler, in his account of “Nordlandene” (the present counties of Nordland and Troms), is the only one to mention the use of fern rhizomes as fodder. Commenting on the mountain above Strete in Gratangen (now Skavlikollen), Troms, he noted that “on the sides, it has the coarse *blomgræs*, of which the root is used for livestock” ([51]: 307). In 1801, Mathias Bonsach Krogh ([52]: 164) noted *Matteuccia* rhizomes among various additional fodders utilized in the Lofoten – Vesterålen archipelago of northern Norway. A few other 19th century records will be cited below.

In the southern parts of Norway, the old tradition of collecting fern rhizomes for fodder rarely survived into the 20th century. Further north, rhizomes were still frequently collected during the early decades of the 20th century, and locally, the tradition survived until World War II or beyond (Table 2).

**Table 2:** Approximate dates of the last time fern rhizomes were collected as fodder at various localities

Locality	Time	Source
Akershus: Ullensaker	c. 1860	NEG 11: 2999
Sogn og Fjordane: Balestrand	c. 1900	[74]:126
Sør-Trøndelag: Leksvik	c. 1880’s	NEG 11: 1728
Nord-Trøndelag: Levanger: Frol	1870’s	NEG 11: 2272
Nord-Trøndelag: Nærøy: Foldereid	1950’s	NFS OAH 519; [4]: 326
Nordland: Bindal	1860’s	[4]: 326
Nordland: Brønnøy	1940’s	NEG 11: 1708
Nordland: Bodø: Kjerringøy	1914–18	[4]: 327
Nordland: Sørfold	1914–18	NEG 11: 1933
Nordland: Hamarøy	1950’s	EBATA 2001:12
Nordland: Sortland	1946–47	[32]: 101
Nordland: Sortland	c. 1945	EBATA 2009:4
Nordland: Bø	c. 1900	NEG 11: 2486
Nordland: Bø	1940–45	[4]: 376
Troms: Harstad	1910	[5]: 378
Troms: Skånland	1940–45	NFS O.A. Høeg 785; [4]: 328
Troms: Ibestad	c. 1900–10	NEG 11: 3413
Troms: Tranøy	c. 1900?	[79]: 187
Troms: Bardu	c. 1900	NEG 11: 2763
Troms: Nordreisa	c. 1935	NEG 11: 4688
Troms: Nordreisa	c. 1940	EBABM 1991:3
Troms: Kvænangen	1930’s	NEG 11: 16672
Finnmark: Alta: Bognelvdalen	1945–50	EBATA 2007:45
Finnmark: Alta: Kviby	c. 1850’s	NEG 11: 22284

At Sortland in the Vesterålen islands, the brief paper of Ingvald Johansen provides a date for the final demise of the tradition, for “as far as I know, my father was the last one to harvest *moldfôr* [here], probably in 1946–47.” ([32]: 101). Locally, rhizomes were still collected in the 1950’s and perhaps even in the 1960’s, e.g. in Hamarøy (EBATA 2001:12).

### Identity – fern species with useful rhizomes

Jens Holmboe was the first to carry out a detailed study of the use of fern rhizomes in North Norway, in particular in the interior valleys of Troms. His brief paper [3] deals with several important questions, i.e. the age and extent of such use, which species were utilized, and which species were avoided – the latter two groups usually reflected in vernacular names. As pointed out both by Holmboe [3] and Ove Arbo Høeg [4], the layman’s species concept for ferns is problematic and highly variable, and it is not easy to identify species from oral traditions alone. Although numerous sources mention the use of fern rhizomes as fodder, very few are sufficiently detailed to allow the species to be identified. The exceptions are records made by botanists who saw or received such material, or interviewed locals, and had sufficient knowledge of fern taxonomy to identify the species. Thus, the exceptions are worth a detailed survey (see also Table 3).

**Table 3:** Fern species recorded as sources of *moldfôr –* rhizomes harvested for fodder use, and priority if several species were used

Species and locality	Vernacular name	Priority	Source
*Athyrium filix-femina*			
Nordland: Brønnøy	storgrofte	1	NEG 11: 1708
Troms: Harstad	mollfor	-	[5]: 378
?Troms: Skånland	mollfor	-	NFS O.A. Høeg 785
*Blechnum spicant*			
Nordland: Vefsn	mållfôr	-	[4]: 327
Nordland: Bodø: Kjerringøy	moltfôr	-	NFS O.A. Høeg 571
*Dryopteris* sp.			
Nordland: Sortland	godmoldfôr	1	EBATA 2009:4
Finnmark: Alta: Bognelvdalen	moldfôr	1	EBATA 2007:45
*Dryopteris expansa*			
Nordland: Hamarøy	moldfôrblom	1	EBATA 2001:12
Troms: Salangen	moldfôr	1	[4]: 328
Troms: Nordreisa	saumoldfôr	1	EBABM 1991:3
*Dryopteris filix-mas*			
Norway	-	1	[45]: 42
Western Norway	maullfoor	1	[46]: 95
Nordland: Brønnøy	storgrofte	1	NEG 11: 1708
Nordland: Vesterålen area	moldfôr	1	EBABM 1990:12
Troms: Balsfjord	telg, moldfôr	2	NEG 11: 19292
Troms: Balsfjord	telg	2	NEG 11: 20628
Troms: Balsfjord: Takvann	moldfôr	1	NEG 11: 21600
Troms: Lyngen	moldfôr	1	[56]: 112–113
Troms: Bardu – Målselv area	moldfôr	1	[3]: 766
Troms: Bardu	moldfôrrot	1	[4]: 328
Troms: Nordreisa	saumoldfôr	1	NEG 11: 4688
Troms: Kvænangen	saumoldfôr	1	NEG 11: 16672
Finnmark: [Alta]	graste	2	[64]: 118
*Matteuccia struthiopteris*			
Nordland: Rana	kujeiske	1	NFS O.A. Høeg 599
Nordland: Sørfold	telli	-	[54]: 48
Nordland: Lofoten – Vesterålen	molfôr	1	[52]: 164
Nordland: Sortland	pil	2	EBATA 2009:4
Nordland: Sortland	storblom	2	EBABM 1990:12
Troms: Harstad	mollfor	-	[5]: 378
Troms: Sørreisa	storblom	1	[4]: 328–329
Troms: Balsfjord	tiske(telj), moldfôr	1	NEG 11: 19292
Troms: Balsfjord	moldfôr	1	NEG 11: 20628
Troms: Balsfjord: Takelvvann	-	2	NEG 11: 21600
Troms: Bardu – Målselv area	telg	2	[3]: 766
Troms: Bardu	blomrot	1	NEG 11: 2763
Troms: Bardu	teljerot, storblomrot	2	[4]: 328
Troms: Nordreisa	kumoldfôr	2	NEG 11: 4688
Troms: Kvænangen	kumoldfôr	2	NEG 11: 16672
Finnmark: [Alta]	maullfor	1	[64]: 118
Finnmark: Tana	-	1	NEG 11: 19729

**Fig. 1:**
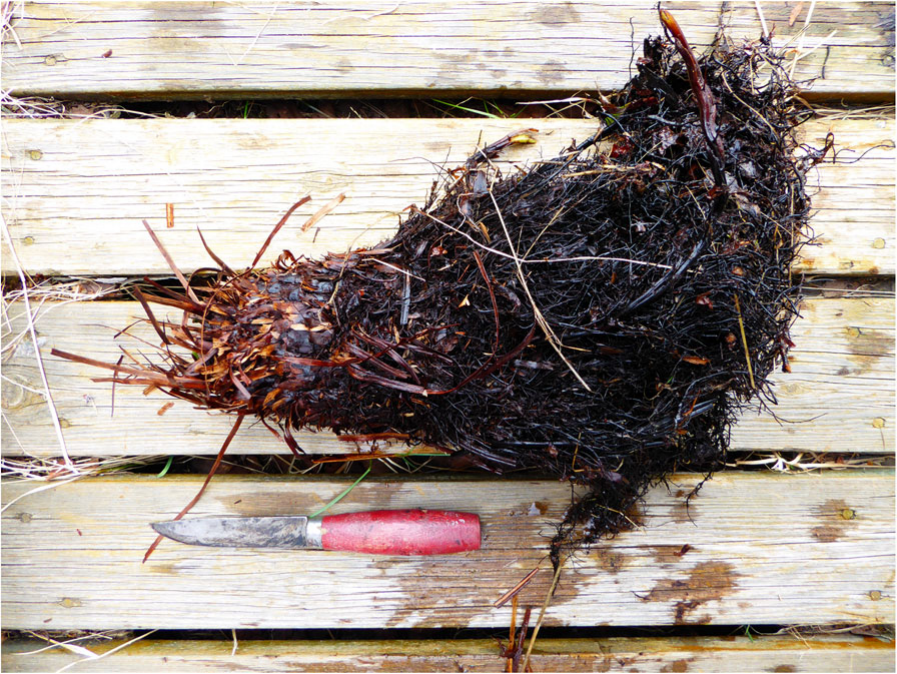
*Matteuccia struthiopteris* rhizome. Although usually considered inferior to the *Dryopteris* species, its large rhizomes have been extensively collected for fodder use

**Fig. 2:**
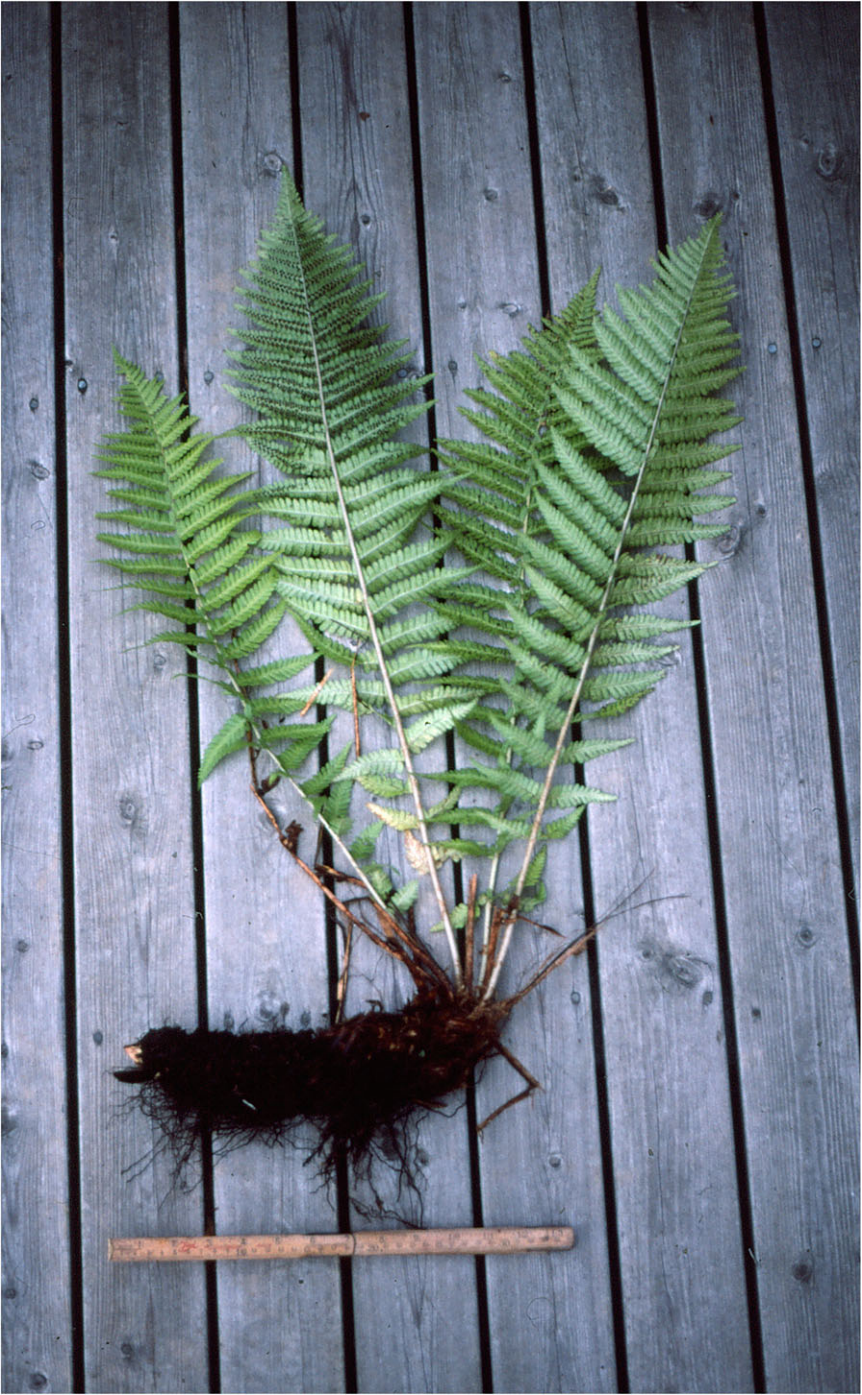
*Dryopteris filix-mas* with its horizontal rhizome. This was a favourite source of rhizomes for fodder use in many areas. (= lysbilde 2000: 1170)

According to the review of Holmboe ([3]: 762), the main species utilized as fodder by the people he interviewed in interior Troms were *Matteuccia struthiopteris* (Fig. 1) and *Dryopteris filix-mas* (Fig. 2), partly also *D. expansa* (called *D. dilata* by Holmboe, but almost all North Norwegian plants belong to *D. expansa*). His material, however, was limited, and later additions tend to modify the picture.

At Balestrand in Sogn og Fjordane, western Norway, the rhizomes of *Matteuccia struthiopteris* were used: “Roots were collected in spring as cattle fodder.” (…) “It was common *strutsvinge* (*Struthiopteris*), which is called *telgblom* there.” (NFS O.A. Høeg 91, 1938; [4]: 326).

A record from the Velfjord area in Brønnøy, southern Nordland, is of particular interest. The informant, Knut Strompdal, was an able botanist, and his identifications are reliable: “Here, ferns are called *grofte*. In spring, when there was little hay, they would chop some *grofterot*. It was the root of *storgrofte* (*Athyrium filix-femina* and *Dryopteris filix-mas*), which people looked for. The root of *einstape* [*Pteridium aquilinum*] was not considered a good root for livestock fodder. I do not know the term *moldfór* from this area.” (NEG 11: 1708, partly cited by [4]: 327).

At Saltdal in Nordland, Axel Hagemann identified the species used as *Matteuccia struthiopteris*: “(…) whereas the roots of the large fern (*Struthiopteris vulgaris*), which grows along the brook in sun-warm slopes, and which is otherwise, under the name of “*brom*” or “moldfor”, given to the catlle to eat, (…)” ([53]: 44; *brom* is probably a printing error for *blom*).

In Sørfold, people would dig up the large rhizomes of *Matteuccia struthiopteris*, called *telli*, for fodder use. They were aware that it could be easily identified by its deviant, brown and overwinterering, fertile leaves ([54]: 48).

**Fig. 3:**
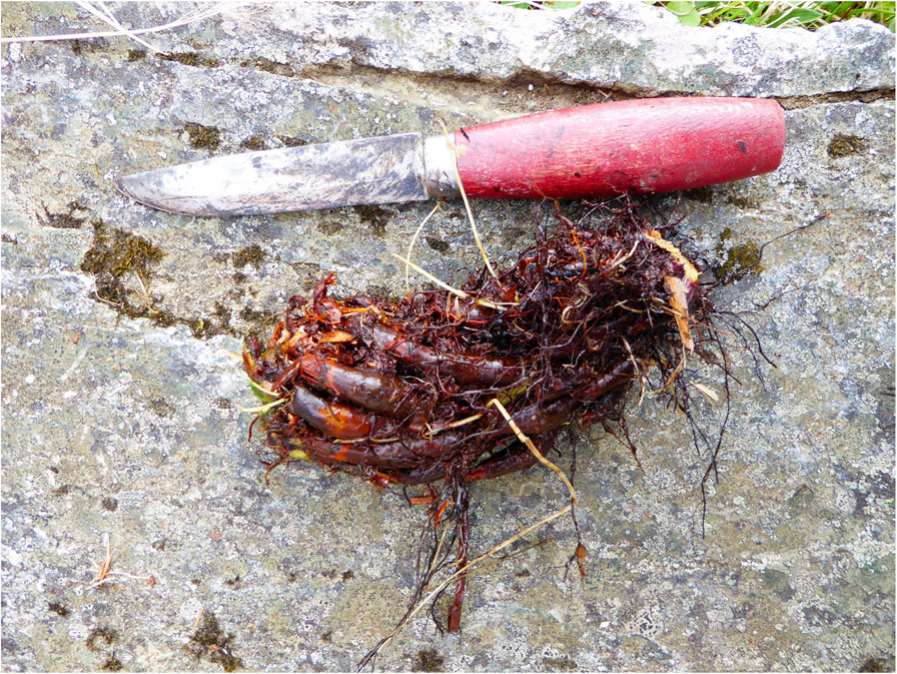
*Dryopteris expansa* rhizome. At least locally, this species was preferred for fodder use, despite its relatively small size (cf. Fig. 3)

At Hamarøy, *Dryopteris expansa* (Fig. 3) was the preferred species, as confirmed by a male informant who had collected fern rhizomes in his youth, and is also an able botanist and thus familiar with the species: “At home it was *sauetelgen* [the official Norwegian name for *Dryopteris expansa*] that was used. And it was called *moldfôrblom*. And the pronounciation was not *mold-*, but *mål-*.» (EBATA 2001:12). In this case, colllection of fern rhizomes had continued into the 1950’s or perhaps even later. He had received instruction from his father how to recognize the desired species: “And so my father said that it was relatively easy to recognize by being, whatever he said, but at least it had these scales along the stem.” (…) “But he said there was one species you could confuse it with, which was rather similar to *moldfôrblomen*, and also had numerous scales along the stem. I am not sure, but I think he must have been referring to *ormetelgen* [the official Norwegian name of *Dryopteris filix-mas*]” (EBATA 2001:12). The latter identification gains support from the sites that were preferred for collecting rhizomes, namely north-facing slopes, often damp and with abundant *Dryopteris expansa*, whereas dry, southfacing slopes, in this area with much *D. filix-mas* and *Pteridium aquilinum*, were avoided. Not least due to need for identifying the correct and desired source or rhizomes, the locals in this area had separate names for several fern species, including *jisk* for *Athyrium filix-femina* and *telli* for *Matteuccia struthiopteris*, collectively known as *storblom* (‘large fern’), whereas *småblom* (‘small fern’) was *Gymnocarpium dryopteris* (L.) Newman, *Phegopteris connectilis* (Michx.) Watt, and perhaps also other species (EBATA 2001:12).

In the Lofoten islands, M.B. Krogh identified *Matteuccia struthiopteris* as the source of fern rhizomes for fodder use: “In addition, the roots of *Osmunda struthiopteris* [= *Matteuccia struthiopteris*] are dug up, and given to sheep and goats, partly raw and partly boiled, and is here called Moldfoer” ([52]: 164).

From Vesterålen in northernmost Norway, a very interesting account is available from a man who had worked as a teacher at an agricultural school for decades. Through his work, he had become acquainted with local practices related to additional fodders, including fern rhizomes. He was also able to identify some of the species used: “In northern Norway, ferns are called *blom*, *småblom* [‘small ferns’] and *storblom* [‘large ferns’]. *Storblom* was, first and foremost, *Matteuccia struthiopteris*. In Vesterålen, *Athyrium filix-femina* is called *pil*, and some called it *blindpil* because if livestock consumed too much of it, they become blind.” He assumed that this was just superstition, because he had never heard of an actual case of such poisoning. According to his experience, the main species utilized for fodder was *Dryopteris filix-mas*: “(…) and this they called *mollfor*, it was frequent in Vesterålen as well.” It was mainly used in spring (EBABM 1990:12).

My own records from the Harstad area in SE Trom suggested that *Matteuccia struthiopteris* and *Athyrium filix-femina* were the main species utilized there ([5]: 378), with the caveat that the male informant’s memory of the ferns he had participated in collecting sixty years ago may have been imprecise. Although distinctly dissimilar to a botanist, *Athyrium filix-femina* and *Dryopteris expansa* may well be confused by the layman; both have finely dissected, pale green fronds. A record from neighbouring Skånland also pointed to *Athyrium filix-femina* (in Norwegian floras: *skogburkne*) as the source of the rhizomes used for fodder: “*Mollfôr* fern roots of *Skogburkne* (…)” (NFS O.A. Høeg 785; 1971). With no specimen provided, or any further information, it is difficult to evaluate if this latter identification was correct or not.

At Salangen in Troms, people used rhizome characteristics to identify the useful and harmful kinds: “It is called *mollfór* when the scales of the root [i.e., the base of the stipe] are round. *Telg* if they are flat. It is only the root that is called *mollfór* or *telg*. All ferns [i.e., the above-ground part] are designated with a common name, *blom*.” (N.F.S. O.A. Høeg 327, ca. 1942; [4]: 328). The description of *telg* is not easy to make out in terms of species, whereas *Dryopteris* spp. have distinctly round and smooth stipe bases, thus fitting the description of the “*mollfór*”.

At Tranøy, fern rhizomes were suggested as a possible means of feeding humans during the hunger years around 1812, with the author identifying the species used ﻿as *Matteuccia struthiopteris*, and noting the widespread vernacular name: “called *Tælg*, i.e. *Tilg*, which no doubt is a species of *osmunda*; the root of *osmunda struthiopteris*, which is here called *Moldfoer* (…)” ([55]: 202).

In 1763–1764, bishop and botanist J.E. Gunnerus in Trondheim received several fern specimens from his subordinates, documenting the species hiding behind various vernacular names – and the source materials of *moldfôr* for fodder use. These remain the only voucher specimens (in herb. TRH) documenting the practice. In April 1763, Gunnerus requested specimens of three different ferns or folk taxonomic units from chaplain Adrian Bødtker in Tromsø (Troms). In due course, he must have received the relevant material, for as noted by Ove Dahl ([56]: 114), the species were identified in the first volume of Gunnerus’ *Flora norvegicia* [43]. Thus, for Tromsø, we know that *Molfoer* and *Lyster Molfoer*, i.e. *moldfôr* and *lystermoldfôr*, derived from *Dryopteris filix-mas*; the naming alone suggests that is was the preferred species for fodder use. *Tælg* (or *telg*) proved to be *Matteuccia struthiopteris*, which may also have been used. The third species, *Teisk* or *blind Teisk*, with is pejorative name, was obviously avoided, and here as elsewhere, the dangerous kind turned out to be *Athyrium filix-femina*. The year after, in 1764, Gunnerus received a specimen of *mollfor* from E.G. Schytte in Lyngen, Troms; it proved to be *Dryopteris filix-mas* ([57]: 289).

Rhizomes of *Matteuccia struthiopteris* have also been collected for fodder, but were considered less valuable than those of *Dryopteris* spp. ([3]: 766). According to Jens Holmboe’s data from Troms, the two kinds were often given separate names. If so, *moldfôr* was reserved for *Dryopteris*, whereas *Matteuccia* was designated as *telg* (see also [15]: 26). At Sortland in Nordland, a recent informant discriminated between *godmollfor* (‘good mollfor’), the best kind, and *pil*, which was *Matteuccia struthiopteris* (EBATA 2009:4). *Matteuccia* was also the prime resource utilized in Sørreisa, Troms: “It was mainly the root of *strussvinge* (*storblom* as it is called here) which was used for fodder. In some places in warm, sun-facing slopes there may be large stands of this fern, with a little *ormetelg* [= *Dryopteris filix-mas*] in between. Thus, it is likely that the root of *ormetelg* was also taken. In our area, people knew the tradition of collection *blomrot* as an emergency fodder. I have participated myself, as a little boy. As far as I know, the practice has now ceased.” ([4]: 328–329). An informant from Bardu responded to the NEG questionnaire by noting that the only kind of root used for fodder was “Ferns (*Strudsvinge*)”, i.e. Matteuccia; “we call it *Blomrot*, which is a large, tall *blom*, which is found mainly in the slopes, or in dense thickets of alder [*Alnus incana* (L.) Moench.] and Euopean bird cherry [*Prunus padus* L.], (…)” (NEG 11: 2763).

The botanist Yngvar Mejland contributed extensively to the NEG archive, providing a number of reports on additional fodders, in which the fern species harvested for rhizomes are identified. A record from Balsfjord is typical: “Now, people have ceased using roots collected in the field. But until the first world war, this was done, and rarely later. In the childhood days of my informants this was still rather common, and in the past (the childhood of their fathers, about a hundred years ago) everyone used roots to feed the animals. It was fern roots that were used. In Balsfjord, ormetelg (Polystichum filix mas) [= *Dryopteris filix-mas*] was called *Telg*. Strudseving (Struthiopteris germanica) [= *Matteuccia struthiopteris*] was called *Tiske*, or *Tisketelj. Moldfor* was a common name. Oddly, in this area the former was considered less good than the latter. Further north – in Lyngen, Nordreisa, Skjervøy and Kvænangen, it was the other way round.” (…) “In Balsfjord, people also said that it was not advisable to feed the animals too much *telg*, because they could get ill. When I asked if this was not due to *trollmoldfor*, which is said to be poisonous, people denied it. To make sure that there was no mistake in the names applied, I went out in the field and collected both *telg* and *strudsvinge*. They were correctly pointed out [by the locals], so the difference from areas further north is real.” (NEG 11: 19292). Mejland received similar information at a second locality in Balsfjord, again with *Matteuccia struthiopteris* identified as *moldfôr*, and the preferred species, whereas *telg* or *Dryopteris filix-mas* was regarded as inferior. (NEG 11: 20628). At Takelvvannet, Mejland’s third locality in Balsfjord, tradition was similar to that further north, and *Dryopteris filix-mas* the preferred source of *moldfôr* for fodder, and *Matteuccia struthiopteris* a less desired alternative (NEG 11: 21600).

From the Nordreisa area of northern Troms, Mejland has contributed an extensive record, identifying the species hiding behind a number of local names. *Blom* was a common term for all ferns; *Dryopteris filix-mas* was known as *saumoldfôr* (‘sheep fern rhizome’), *Matteuccia struthiopteris* as *kumoldfôr* (‘cow fern rhizome’), and *Athyrium filix-femina* as *trollmoldfôr* (‘troll fern rhizome’), the latter obviously pejorative and serving as warning: “You had to be cautious with *trollmollfór*, because it was poisonous. It was identified by the spines at the base of the leaf stem.” (…) “*Saumollfór* was the best kind. I have seen myself that the cows would take it first from a mixture with *kumollfór*” (NEG 11: 4688).

The practice in neighbouring Kvænangen was much the same: “The ferns people collected were *ormetelg* [*Dryopteris filix mas*] = *saumoldfor* and *Struthiopteris germanica* [*Matteuccia struthiopters*] = *kumoldfor*” (NEG 11: 16672). According to Mejland’s note, people had to avoid *Aspidium spinulosum* [= *Dryopteris carthusiana* (Vill.) H.P.Fuchs), but this may be an error – the species is rare in Troms. He goes on to note that people recognized the harmful kind by the spines at the base of the stem; the layman’s preferred character to single out *Athyrium filix-femina*. “People told me about poisoning caused by it. It was first noted by [the] animals starting to swagger, and their hind part became lame, so that they were sitting. In the worst cases, they had to be slaughtered.” (NEG 11: 16672). At Eibydalen in Alta, Finnmark, the effect of poisoning was described in similar terms: “If the animals ate much *trollmuldfor*, their hind legs were lame, and they were sitting in their pens – and could die.” (NEG 11: 21066).

An early identification of the useful fern species is found in country prefect Ole Hannibal Sommerfelt’s topographical description of Finnmark, Norway’s northernmost county, in 1799 ([58]: 118). He lists some of the plant species found there (see review in [59]), including “*Maullfor* og *Graste* (Osmunda struthiopteris and Polypodium filix mas), of which the two latter kinds produce near the root thick, inside (when they have been broken apart) cabbage-green leaves in the form of an artichoke, which, when they are boiled and pulled apart, give the livestock a well-tasting fodder.” Sommerfelt’s account thus also singles out *Matteuccia struthiopteris* and *Dryopteris filix-mas* as the main sources of fern rhizomes for fodder use, seemingly identifying *Maullfor* with the former, and *Graste* with the latter. *Graste* is perhaps just a misreading of Sommerfelt’s manuscript, approaching the more well-known term *Grofte*, which would look very similar in Gothic script. In all likelihood, he based his account on local tradition in Alta, where he resided.

In Tana, only *Matteuccia struthiopteris* was present in sufficient quantity to yield much in terms of fodder: “Fern roots have been used to some extent, in particular *Struthiopteris germanica* [= *Matteuccia struthiopteris*]. *Ormetelg* [= *Dryopteris filix-mas*] is very rare in Tana.” (NEG 11: 19729).

Two records from Nordland, both included in Ove Arbo Høeg’s material, deviate from the above pattern by suggesting that *Blechnum spicant* served as a source of rhizomes for fodder use. The records derive from coastal areas in Brønnøy and Bodø: Kjerringøy (NFS O.A. Høeg 571; 1948), where the species occurs abundantly, and utilization cannot thus be ruled out, despite the fact that its rather small rhizomes suggests that collecting material would be an ardous task. Høeg cites only the former record in his published compilation ([4]: 375).

**Fig. 4:**
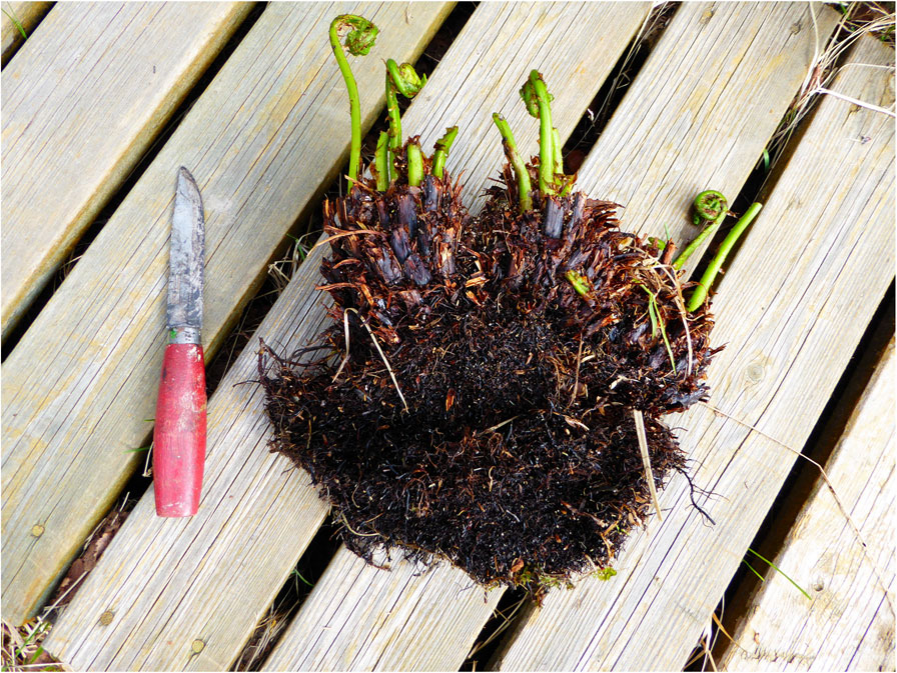
*Athyrium filix-femina* with rhizome. This species was usually avoided, and designated by various pejorative names. It was considered harmful to livestock, and recognized by the spines or protuberances at the base of the stem

**Fig. 5:**
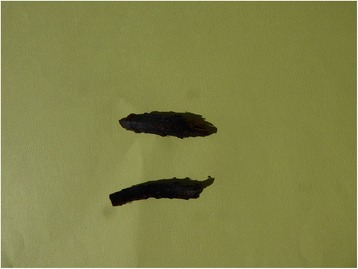
Basal part of the petiole of *Athyrium filix-femina*, showing the spines referred to in folk tradition as identifying the harmful kind of *moldfôr* or fern rhizomes, generally rejected as fodder

The records cited so far agree that the rhizomes of the presumed harmful species could be identified by the protuberances or spines at the basal part of the stem – which clearly points to *Athyrium filix-femina* (Figs. 4 and 5). A brief note on *moldfôr* at Andøya in Andøy, Nordland seemingly deviates from this pattern, but what is called “spines” should probably rather be read as “stipes”: “But we reckoned there were two kinds of *moldfôr*. Some had round spines/knobs on the tuber, and it was these we used. The second kind had flat spines, and these we must not use. It was called *blindmoldfôr*, and it was said that the sheep became blind it they ate it. It was said that this [condition] only lasted for a short while. After some time, their sight was restored.” ([60]: 55).

With a few exceptions (e.g. at Brønnøy in Nordland, cf. above), folk tradition in northern Norway is uniform in singling out *Athyrium filix-femina* as poisonous and harmful (see Table 4). Surprisingly, negative effects of using *Dryopteris* spp. rhizomes for fodder were little known, despite the fact that *D. filix-mas* is a well-known and age-old remedy for intestinal parasites, and thus obviously poisonous. In human beings, large doses may cause lethal poisoning ([3]: 767, [61]: 335–338); see also discussion.

**Table 4:** Species avoided when collecting fern rhizomes for fodder us

Species and locality	Vernacular name	Source
*Athyrium filix-femina*		
Nordland: Hamarøy	jisk	EBATA 2001:12
Nordland: Vesterålen area	pil, blindpil	EBABM 1990:12
Troms: Bardu	teskrot	[4]: 328
Troms: Nordreisa	trollmoldfôr	NEG 11: 4688
Troms: Kvænangen	-	NEG 11: 16672
Finnmark: Alta: Eibydalen	trollmoldfôr	NEG 11: 21066
Unidentified, but probably *Athyrium filix-femina*		
Nordland: Sortland	blindmoldfôr	[25]: 26
Nordland: Sortland	pilmoldfôr, pil, blindpil	[25]: 26
Nordland: Hadsel	blindmoldfôr	[25]: 26
Nordland: Hadsel	pilmoldfôr, pil, blindpil	[25]: 26
Nordland: Vågan: Gimsøy	blindmoldfôr	[25]: 26
Nordland: Bø	blindmoldfôr	[25]: 26
*?Dryopteris filix-mas*		
Nordland: Hamarøy	-	EBATA 2001:12
Troms: Målselv: Dividalen	mollfôrrot	EBABM 1989:5–6
*Pteridium aquilinum*		
Nordland: Brønnøy	-	NEG 11: 1708
Nordland: Hamarøy	moldforblom	EBATA 2001:12

In some further cases, noted below, the species used may at least be hinted at, based on the details available.

### Harvesting fern rhizomes – and tools used

Various tools were used to dig up or extract the rhizomes, most frequently a hoe or a spade, or sometimes a knife. At Velfjord in Brønnøy, Nordland, people seemingly had no particular preference in this respect: “It was dug up with a hoe or a pick, and sometimes a spade could be used.” (NEG 11: 1708). In Steigen, fern rhizomes “were picked up with a hoe and brought home in sacks and stored in large piles in a shed.” (NEG 11: 1644). At Vågan in Lofoten, “they used a hoe to dig it up” (NEG 11: 2463). At Andøya in Vesterålen, a knife was used to extract the rhizomes: “(…) they were cut off with a knife, and then dried, before being used as additional fodder.” ([62]: 13). At Hadsel, further south in Vesterålen, a ruined scythe was reused for this purpose, to make a *moldfórkniv* (‘fern rhizome knife’): “As to the knife in question, it was made from a broken scythe, broken in the middle as shown by the drawing. It was the pointed end that was used. At the hind end, a rag was coiled to avoid harming the hand. One used the left hand to grab the fern at the base, and with the right hand, one would insert the knife in the soil, and turn it around the base to cut the roots, which are well attached.” (NEG 11: 1111; partly cited by [4]: 327). A record from Øksnes is less detailed, saying only that “They prepared special utensils which were used to pull up the *mollfor*.” (EBABM 1990:10; [63]: 14).

Ingvald Johansen provides a detailed account of how the harvest of fern rhizomes was carried out at Sortland, also in Vesterålen: “The fern grows in slopes with a thick soil. In the mountain slope between [the lakes] Skyggevatnet and Durmålsvatnet, there was an abundance of this plant, and in the past, a lot of *moldfôr* was taken here in the autumns. In this area, it was mostly people from Kjerringvik and Valfjord who carried out the harvest [of rhizomes]. The tool they used, was a long knife. One made a cut around the root, grabbed the plant, and pulled the *moldfôr* out of the soil. It was slow and painstaking work, and it was often carried out for several days. When the day’s work was completed, one collected the rhizomes in sacks and carried them to [the boulder called] *Moldfôrsteinen*. Here, they were poured out of the sacks, and heaped in piles [and stored] until the snow appeared. There was no need to cover the store. When the ground was covered by snow, the *moldfôr* was fetched with a toboggan or sledge” ([32]: 100).

In Harstad, Troms “they used a hoe” ([5]: 378). Also in Harstad, an old and worn-out knife at my grandmother’s farm was referred to as a *moldfôr* knife – “but I didn’t know what it was.” “It was truly a *moldfôr* knife, they said” – presumably to suggest that it could no longer be used for anything else ([5]: 378). At Balsfjord, further north in Troms, a hoe was used: “Fern roots were chopped with a hoe and carried or driven to the sea. One could place them in bags on the sledge, but some had a frame.” (NEG 11: 20628). A hoe is also mentioned in a second record from Balsfjord (NEG 11: 17292), and from Nordreisa (NEG 11: 4688).

Thus, in general, some kind of knife or other utensil was used to cut the *moldfôr* or rhizomes loose. No matter what kind of tool people used, extracting the rhizomes usually required some digging, which is certainly necessary e.g. for *Dryopteris* ssp. The only exception to this rule seems to be *Matteuccia struthiopteris*, where a substantial part of the rhizome may protrude above ground. A man from Sortland in Nordland noted that it could be collected by a deviant method: “Referring to that *pil …* They collected it later in the year, when the ground was frozen. Then, it was almost above ground. They hit it with a club” – and this was sufficient to get it loose; there was no need for digging it up. (EBATA 2009:4).

### Timing of harvest and use

Limited information is available on the time of year when rhizomes were collected. According to Jens Holmboe, the work was frequently done in autumn, and the rhizomes stored in piles until need arose – usually in late winter or early spring ([3]: 765). The rhizomes were then brought home, and the cattle fed, sometimes with raw rhizomes. More frequently, they were boiled together with a varied recipe of other additional fodders, e.g. in Finnmark including birch twigs, fish remains, and even horse dung ([64]: 140).

At Leksvik in Sør-Trøndelag, fern rhizomes were collected at the end of the season: “They were usually collected in late autumn, just before the snow.” (NEG 11: 1728). Knut Strompdal’s record from Velfjord in Brønnøy, Nordland provides a detailed account, referring only to collection in spring: “The roots were not stored for any length of time, [but] they could be left standing in a box or some other container until they were used. I have never heard of anyone collecting *grofterot* in the autumn.” “In the autumn, people did not collect *grofterot*. This may be due to the late cessation of other autumnal work; when completed, the frost usually appeared, and perhaps even snow. Therefore, the roots had to be taken in spring.” (NEG 11: 1708). The record also provides details on use: “*Grofterot* was considered an additional fodder. The rhizomes were supposed to have substantial value as fodder, but were otherwise not highly regarded as far as I have heard, and as fodder, it was not accepted by all the animals. When used, they were chopped into small pieces, and preferably served along with kitchen refuse or with fish remains. It was mainly the cows who were served *grofterot*.” (NEG 11: 1708; cited in [4]: 327).

At Elsfjord in Vefsn, Nordland, people collected rhizomes “in spring” (NFS O.A. Høeg 719; 1956), whereas the harvest in Beiarn could be carried out both in spring and autumn: “Some would dig up the roots in the autumn and let [them] lie outdoors, but most of it was dug in spring and used as additional fodder for the cows.” (NFS O.A. Høeg 793; 1971).

In Sørfold, people would row to a certain slope that had abundant stands of *telli* or *Matteuccia struthiopteris*, and bring the rhizomes home. Their handling was simple: “They were placed in luke-warm water, washed clean, and then carved up and given to the livestock” ([54]: 48).

In Steigen, rhizomes were served to all kinds of livestock: “These were given to all animals, even horses, in their raw state. It was considered a fine additional fodder for the livestock; even the pig ate the *moldfor*” (NEG 11: 1644). Another record from Steigen is more restricted in terms of the recipients: “*Mollfór*, the root of larger ferns, for goats and sheep.” ([4]: 327).

People at Hamarøy, further north in Nordland, would only search for fern rhizomes when need arose, i.e. when they were running out of hay and other fodders: “Collected in spring. The roots [rhizomes] were dug up and served directly to the animals in their pens.” (EBATA 2012:16).

A record from Ballangen in northern Nordland reports collection both in spring and autumn, and is unusual in suggesting that the work was carried out by females: “During the spring fodder shortage, one went into the forest and dug up fern roots. It was called *moltfòr*.” (…). “From the very start of the autumn the women were up in the *moltfòr* slope digging roots for the sheep, the small animals, because the fodder collected during the summer was insufficient.” ([31]: 77).

A number of records from the Lofoten-Vesterålen area states that rhizomes were collected in autumn, e.g. at Sortland: “*Moldfór* was collected in the autumn. It was usually boiled before it was used. The decoction had a finer scent than that of hay.” ([4]: 327). A second record from Sortland provides some additional details on rhizome harvest: “They had to collect them in autumn, before the ground was frozen.” (…) “They used to pile them up in the outfield areas, and pull them home on snow-covered ground. It was chopped up and used for the cattle. It was considered a good fodder, not an emergency fodder, but a good additional source.” (NEG 11: 2486).

At Øksnes in Vesterålen, the collecting work was seemingly done in late summer or early autumn: “(…) it was a whole work season. People would scythe the outfield and infield areas. Afterwards, they brought home the *mollfor*, which they stored in the barn.” The informant himself had not participated, but knew the practice through his father (EBABM 1990:10; [63]: 14).

A record from Skånland in SW Troms provides some further details in terms of how the rhizomes were handled: “They used it a lot in spring, [it] was crushed with the back of an axe, and parboiled, often together with fish offal.” (NFS O.A. Høeg 785, 1971; [4]: 328). In nearby Ibestad, people collected the rhizomes at the end of the season, using them as fodder throughout the winter season: “It was mainly in late autumn people dug up the roots. In spring, the soil was frozen.” (…) “The fern roots were stored in the entrance to the barn, and used as fodder in the course of winter” (NEG 11: 3413). At the major island of Senja (Vangsvik in Tranøy), harvesting traditons were much the same: “Here, people have used *blom*. The roots were cut in the autumn, and carried home in a sack. One could also chop roots and leave them well into spring” (NEG 11: 22600).

Yngvar Mejland provides an account of traditional harvesting in the Bardu – Målselv area of interior Troms: “In the autumn, people could cut large piles of roots [fern rhizomes], which were brought home in winter. From slopes far away, the roots were transported home on the snow crust in spring. People had to be aware of and avoid *trollblom*, which was poisonous.” If need arose, rhizomes could also be collected in spring: “It frequently occurred in spring that people had to bring a spade to shovel away the snow to find the roots.” “Previously, large quantities of *blom* were used here.” (NEG 11: 22561).

A record from Bardu is related to the former use of *blomrot* or *Matteuccia struthiopteris* (see below), but is more detailed in terms of the timing and mode of use: “It is mainly in spring, when the snow thaws in the slopes, and there is a fodder shortage, that people would use *blomrot*. But the root was also collected in the autumn, and placed in round piles. But this practice is now long since obsolete, but before and around 1900, and a little later, *blomrot* was used. It was chopped into pieces and mixed with seeds from the hay, light grain, awns and litter, a little horse dung, and perhaps some fine rowan bark, and a little salt was fine.” The whole mixture was boiled and served to the cattle. (NEG 11: 2763, partly cited in [4]: 328).

A record from Balsfjord points to rhizome collection in late spring: “Sprouting roots were previously used as fodder for cattle, and called *moldfórhovver*.” ([4]: 328). In this area, a hoe was used for collecting rhizomes: “The roots were cut with a hoe, and placed in piles, or put in a sack at once and taken home. People would beat it to get rid of the soil, and give the entire roots to the animals. Large roots could also be chopped into pieces. *Moldfor* was considered a very good fodder.” (NEG 11: 19292).

Yngvar Mejland’s detailed account from Nordreisa (Troms) provides details on this as well: “The roots were cut loose in the autumn, with a hoe, and placed in heaps. Sometimes, one would also clear away the snow in winter, and axe out roots from dense stands. In spring as well, when the snow disappeared from the slopes, one would cut *mollfór*.” (NEG 11: 4688).

In Alta, Finnmark, an 18th century record provides an exact date for the intended collection of fern rhizomes: “At September 9, 1776, some of the local inhabitants at Langfjorden visited Langfjordbotn to collect Moldfoer (roots of ferns)” (…)” ([65, 66]: 22). They went into the valley leading over to Alteidet in Troms; an area with abundant stands of *Matteuccia struthiopteris*, which was thus probably the species they intended to harvest. The intention was not fulfilled; instead, they turned into a small band of foreign criminals, one of whom was shot and killed, leading to a trial and court documents recording the event (see also [67]). At Eibydalen, further east in Alta, farmers of Norwegian and Finnish ethnic origin waited until need arose: “The roots were cut in spring” (NEG 11: 21066). In Tana, much further east, people made an effort at collecting fern rhizomes at the end of the summer: “They were cut in the autumn. People considered them to be a fine fodder. They were carried home at once, or stored in a pile and fetched during the winter.” (NEG 11: 19898).

It rhizomes had been harvested in any quantity in the autumn, they would usually be brought home in winter, when the ground was covered by snow. A man from Sortland in Nordland had participated in this part of the process during his youth: “I can remember it from my childhood.” The harvesting as such was done by his father. “I only participated in dragging it home in winter.” (EBATA 2009:4).

Most toponyms incorporating the term *moldfôr* (see above) undoubtedly refer to sites with large fern stand, in particular areas where rhizomes were collected. Exceptions occur, e.g. in Sortland, Nordland, where a boulder got its name from serving as a place to store rhizomes: “In the slope above “Dalstua” [a cottage] in the Holmstaddalen valley, there is a large boulder. It is about two meters high, and its lower side is slanting inwards, thus forming a hiding place. It was called *Moldfôrsteinen* [‘the fern rhizome boulder’], and it was a well-known landmark to people at Kjerringvika and in Valfjorden. Everybody was familiar with *Moldfôrsteinen*, for beneath it, the *moldfôr* was stored until the snow came. Nowadays, few if any know *Moldfôrsteinen*. They know nothing of the *moldfôr* either.” ([32]: 100).

Hunger-feeding of animals during late winter and spring was common in much of Norway at least until the end of the 19th century. Caspar Holten Jensenius, an agronomist visiting Finnmark in the mid-19th century, made a typical note on local practices: “I was told that people in many places thought it sufficient, if fodder would last until Candlemass [2 February]. The rest of the winter, the livestock would have to survive on fish remains, kelp, seaweed, *Moldfoder* (the roots of a kind of fern), heather, twigs, and the sort” ([68]: 6; cf. [69]: 86).

Fern rhizomes were mostly resorted to as fooder in spring, if a long winter or insufficient supplies of hay lead to shortage. A typical record is available from Lavangen in Troms, referring to *mollfor* from *blom*: «In spring, where fodder was in short supply. It was chopped apart and given directly to the livestock” (EBATA 2007:79).

The cattle did not object to fern rhizomes. In summer, they could in fact sometimes tear up such rhizomes themselves ([4]: 765), as had also been noted at Elsfjord in Vefsn, Nordland: “Otherwise, the cows take them, when and where they can find them.” (NFS O.A. Høeg 719; 1956). Goats could also dig up the rhizomes in spring [3]. This had been noticed at Rollag in Buskerud as well: “She also said that the goats were so fond of the fern roots in the autumn, they would chew everything they were able to access of it” ([70]: 10). Further north, people had noted that wild animals could do the same thing, e.g. moose *Alces alces* (L.), as recorded in Sørfold, Nordland: “After the moose appeared in the area not long ago, it was noted that that it also appreciated *moltfôr* as winter food.” ([71]: 82). People in Bardu, Troms, confirmed this observation: “Otherwise, people had noted that the moose was very fond of *telgrot*, *storblomrot* [= *Dryopteris* spp., *Matteuccia struthiopteris*], especially in winter.” ([4]: 328). People in neighbouring Målselv had noted the same: “I only known that the moose will dig up and eat roots” (EBATA 2010:41).

People’s opinion as to the quality of fern rhizomes as fodder varies considerably, perhaps to some extent reflecting the fact that different species were used. At Leksvik in Sør-Trøndelag, the verdict was less than enthusiatic: “These roots were dry, with little juice, and were not considered a good fodder. It was not used for horses, but mainly for cattle, and only in times of need.” (NEG 11: 1728). A little further north, in Levanger, people found them much better: “It was a very good fodder.” (NEG 11: 2272).

In Lofoten, fern rhizomes were served to a variety of domestic animals: “People used is as an additional fodder for the livestock, including pigs, from what I have heard.” (NEG 11: 2463). Ingvald Johansen’s account of the use of fern rhizomes at Sortland in Vesterålen provides some details on the way it was used to feed livestock: “The goats and sheep in particular were fond of *moldfôr*. For them, the roots were chopped up or torn apart. Three or four roots were a good supplement to the fodder, and allowed a substantial saving in terms of hay. The cows also enjoyed eating *moldfôr*. At that time, one would usually attend the barn three or four times a day. If people had fish heads or other fish refuse, they were cooked for use with the midday fodder.” (…) «In our home, feeding with *moldfôr* was done in this way: For the sheep, the rhizomes were chopped into small pieces and placed dry in the manger. For the cattle, one would heat water in the kitchen. The *moldfôr* was chopped up, some four or five rhizomes. They were placed in a bucket, and hot water poured over them. On top of this, half a kilo of fish (herring) flour was strewn. The animals were treated well, and they were pleased with this serving. One could of course also give the cows *moldfôr* in its dry condition” ([32]: 100).

In interior Troms, fern rhizomes were also used as fodder in the autumn, before the slaughtering season. According to Jens Holmboe, fronds were sometimes also used. People considered fern rhizomes a useful fodder at this time of year; it was supposed improve the meat of cattle, and to make pork taste better ([3]: 765).

In the far northeast of Troms, at Kvænangen, rhizomes were collected both in autumn and spring. “One would dig up the fern root in the autumn, and partly in spring. As long as the supply was sufficient, it was a frequent source of fodder, and it was considered a fine fodder. If people collected a large quantity, it was left in the shed until it was used.” (NEG 11: 16672). The rhizomes served mainly as cattle fodder: “One would collect the *moldfor* in the slopes, and carry it down to the sea, and row it home. The roots were crushed and given raw, mainly to the cows. The cow had to get the best fodder” (NEG 11: 16672).

The nutrient value of fern rhizomes has been analysed twice. Ove Arbo Høeg ([4]: 329) cites a 1940–41 study of *Dryopteris filix-mas* rhizomes, carried out by Anders Lothe. Twenty years later, Karl Fjærvoll had rhizome samples of both *D. filix-mas*, *D. dilatata* (which would now rather be named as *D. expansa*), and *Matteuccia struthiopteris* from northernmost Troms analysed ([72]: 306). The results of both studies are very similar; Fjærvoll concluded that the fodder value of the rhizomes was comparable to hay of *Phleum pratense* L.

#### Unidentified fern rhizomes as fodder

With the exceptions outlined above, most records available on the collection and use of fern rhizome do not provide sufficient details to identify the species used. Thus, they only attest to the wide-spread and long-lasting Norwegian tradition of collecting fern rhizomes for fodder. They do, however, provide a wealth of information on the practice in general, useful e.g. for mapping its geographical distribution. A selection of these sources are compiled and quoted below.

**Fig. 6:**
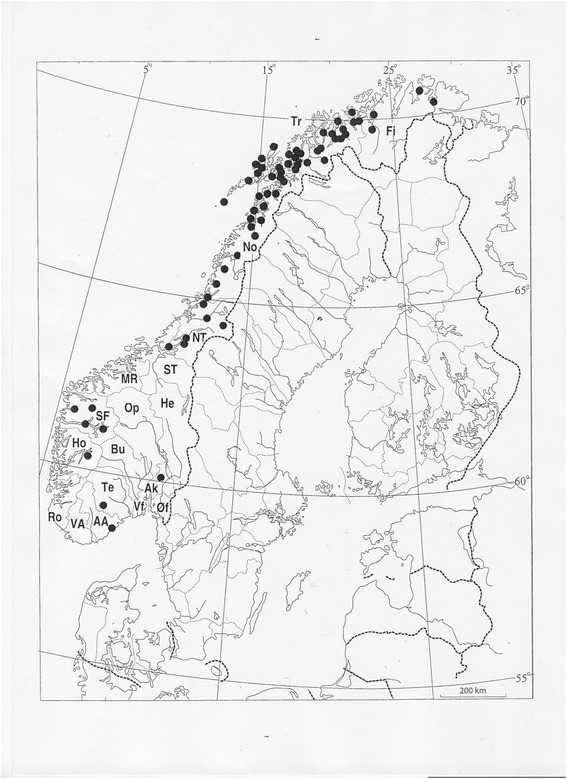
Geographical distribution of the tradition of collecting fern rhizomes for fodder in Norway, compiled from a variety of published and unpublished sources. Two-letter codes indicate the main administrative units (counties): He Hedmark, Op Oppland, Øf Østfold, Ak Akershus, Vf Vestfold, Te Telemark, AA Aust-Agder, VA Vest-Agder, Ro Rogaland, Ho Hordaland, SF Sogn og Fjordane, MR Møre og Romsdal, ST Sør-Trøndelag, NT Nord-Trøndelag, No Nordland, Tr Troms, and Fi Finnmark. The small area of Os Oslo is included in Ak

As seen from the map (Fig. 6), the tradition of harvesting fern rhizomes for fodder is found along the entire west coast of Norway, i.e. in coastal areas with a humid climate that promotes the growth of ferns. Records of such use are generally few and far between in the southern part of the country, and show a marked concentration to the three northernmost counties (Nordland, Troms, and Finnmark). This pattern is confirmed by the NEG material (Table 5). Informants in the southern part of Norway invariably reported having no knowledge of fern rhizomes as fodder, and the term *moldfôr* was unknown to them. By the mid-20th century, fodder use had seemingly mostly been forgotten in the two counties of Trøndelag (central Norway) as well, whereas a majority of the informants in Nordland and Troms confirmed the practice, often from their own experience, thus replicating the pattern of Fig. 6, and clearly demonstrating the tradition’s strong concentration to northern Norway.

**Table 5:** County-wise distribution of mid-20th century knowledge of the use of fern rhizomes for fodder, according to the NEG material (number of records mentioning use, or absence of such). For a map of the counties, see Fig. 6

County	Used		Not used	
	Records	%	Records	%
Hedmark	0	-	11	100
Oppland	0	-	2	100
Østfold	0	-	1	100
Akershus	0	-	3	100
Buskerud	0	-	5	100
Vestfold	0	-	7	100
Telemark	0	-	8	100
Aust-Agder	0	-	9	100
Vest-Agder	0	-	10	100
Rogaland	0	-	22	100
Hordaland	0	-	24	100
Sogn og Fjordane	0	-	13	100
Møre og Romsdal	0	-	12	100
Sør-Trøndelag	1	7.1	14	92.9
Nord-Trøndelag	2	20.0	8	80.0
Nordland	11	57.9	8	42.1
Troms	14	92.9	1	7.1
Finnmark	5	50.0	5	50.0
Sum	33		163	

#### Akershus

The NEG survey provides a single record of former use in Ullensaker. The informant reported that “fern [rhizomes] were collected, if only locally, in autumn, and given to the sheep.” He could not remember what they were called. Such use was long since obsolete: “far back in time, about 1860 I think, but my father probably used it around that time”. Local supplies were restricted, and the rhizomes were only considered an inferior kind of fodder (NEG 11: 2999).

#### Telemark

A single record of former use derives from Fyresdal: “The roots were collected in spring. During later years, this has been very rare, and such use has now ceased.” ([4]: 326). Rhizomes may also have been collected in Kviteseid, although the fronds were more important: “One heard less about the use of the roots.” (NEG 11: 2773).

#### Aust-Agder

Fern rhizomes have been collected in Arendal (Flosta): “In the past, people gathered roots of a fern species which is called *burot* here. It is *ormetelg*” [= *Dryopteris filix-mas*]”. The informant identified it as “*Ormtegl*”, i.e. *Dryopteris filix-mas*, but the description given of its habitat (“shaded, damp sites”) does not really fit (NFS O.A. Høeg 491; 1956; see [4]: 326).

#### Hordaland

Only two records are available, and they are separated by half a millennium. The first one derives from Hardanger in 1283 (cited above), and the second from Ullensvang, also in Hardanger, Hordland. According to the latter, the term *moldfôr* was still remembered in the 20th century, but nothing is said in terms of collection and use: “There was a lot of *mòlfor* i Trettesriplann ([73]: 69).

#### Sogn og Fjordane

Rhizomes as fodder are noted from the Sunnfjord area around 1800: “*Tælgroden* is here regarded as the very best means of compensating for the lack of fodder, bus as far as I know, it is only found in a few places.” ([42]: 88). People in Balestrand collected the rhizomes of *telglbom* or *Matteuccia struthiopteris*: “We have [a place called] Telgskrida, where the *telg* is growing, and the verb *telga*, to collect *telg*.” (…) “They cleaned the roots and gave to the cows.” (NFS O.A. Høeg 91, 1938; [4]: 326); “They also used the root of *telg* for fodder.” (NFS O.A. Høeg 410; 1944). In this area, rhizomes were still collected in the early 20th century: “Sometimes, they were up in the screes and dug up the roots of *telgblomen* and gave to the livestock.” ([74]: 126). Two other records provide no identification of the species used, e.g. in Jølster: “In the autumn, [and] in winter as well, if need be, they collected both the *blom* [i.e., the fronds] and its root, which they soaked in water and gave to the livestock.” ([4]: 326). The record from Lærdal is less detailed, and somewhat misleading in terms of the part used: “*Telg* meant the spring growth of the *blom* which they collect in spring for cattle fodder” ([4]: 326).

#### Sør-Trøndelag

The NEG material comprises a single positive answer, from Leksvik: “Fern rhizomes (*blom*) were also collected and used for fodder in our part of the countryside some 70 or 80 years ago. Since then, this additional fodder has not been used – at least not to any extent. There were two or three different kinds of fern – *blom – storblom* [large fern], *småblom* [small fern] and bjørnekam [*Blechnum spicant*]” (NEG 11: 1728; partly cited in ([4]: 326).

#### Nord-Trøndelag

Available records are more extensive for this area, though none of them provide any identification of the species used, e.g. at Foldereid: “In Bjørågrenda, they are still using fern roots, *grefte*, as fodder, and it is not considered an inferior kind of additional fodder.” (…) “It is collected in spring. There are two localities here called Greftesli.” ([4]: 326). At the other sites, the use had ceased further back in time, e.g. in Levanger (Frol): “My mother said that in her youth, about 1875, they collected “*Jeiskrot*” (fern roots), and used it to fatten goats in the autumn. They had also used it for pigs.” (NEG 11: 2272, also cited by ([4]: 326). Use at Grong was governed by local availability: “The only thing I have heard, is that up in the Sandørdalen valley, they collected *grofterot –* fern root – and used it as an additional fodder for cattle. Further down in the settlement, ferns are so sparse that it was not possible to use them as fodder.” (NEG 11:1740, cited in [4]: 326, with some misreading). A note from Sparbu merely states that: “*Jiskerot* was used for fodder “([4]: 326). A record from the inland area of Nordli in Lierne is more extensive: “In many places, they would go digging for *grøftrot* in early spring. In the Gufjellet mountain, the southern side was early bare in spring. Long before the ice disappeared on the lakes, and the infields got green, the slopes at Gufjellet were green. Up in the mountain, there were *grøften* and *turt* [= *Cicerbita alpina* (L.) Wallr.]. They went across the ice to Gufjellet and fetched *turt* and *grøftrot* for cattle fodder. *Grøftrota* was about the size of a closed fist. They put it in sacks. It was very helpful. They could have a whole load of it. The cattle were very eager. In spring, they saved the cattle with it. *Grøfterota* was their rescue.” ([4]: 326).

By far the most extensive use of fern rhizomes in Norway is found in the three northernmost counties: in Nordland, where “The fern root or *molfor* has long been known and priced as fodder” ([75]: 598), Troms, and Finnmark. For the latter, Amund Helland noted in 1905 that: “Another additional fodder is the so called *moldfoder*, which is the root of ferns. It is considered a good fodder (…)” ([76]: 372). A single record in Ove Arbo Høeg’s material mentions such use from scattered municipalities within this area: “*Blomrot*, roots of *ormetelg* [*Dryopteris filix-mas*] etc., was previously often collected as fodder in Sørreisa, Bardu, Målselv, and at Værøy.” ([4]: 327–328).

#### Nordland

Fern rhizomes have been collected throughout the county, e.g. at Bindal in the far south: “When there was a shortage of fodder, they collected *grofti* roots. This ceased in the 1860’s. A man said that when he was a small boy, he had to go up in the slopes to collect *grofti* roots as soon as the land got snow-bare in spring. It was an unpleasant task.” ([4]: 326). In Vefsn, people utilized an unidentified fern, called *grøfte*, but also partly resorted to *bjønnkam* or *Blechnum spicant*: “*Grøfte* and *bjønnkam* were used as *mållfór*.” ([4]: 327). The species used in Rana may have been *Matteuccia struthiopteris*: “*Kujeisken* is the large, feather-shaped fern. It sprouts from a stout, black root neck, which was previously used as an additional fodder during the springs.” (NFS O.A. Høeg 499, 1940’s; [4]: 327). An extensive record from Brønnøy has been cited above. It refers mostly to past use, noting that “Nowadays, *grofterot* is hardly used. Perhaps a little is collected at some places in lean years. The last war [World War II] made no difference. *Grofterot* was not used more then than is otherwise the case. Previously, it was supposedly more frequently used, but not every year. It was used as an additional fodder during the spring shortage. This has been the case as far back in time as people can remember.” (NEG 11: 1708).

Rhizomes were also used in Beiarn, Nordland: “*Blôm* was also called *målljfór-blom*, because the roots [rhizomes] were used as cattle fodder.” ([18]: 138). Here as well, local availability governed the extent of such use: “In the old days, people would use everything that could support their livestock as fodder, and where it was possible to find various kinds of roots, these were utilized. However, the availability of roots differed a lot, in particular for ferns. Beiarn has received settlers from many areas of Norway. The upper part of Beiarn, with side valleys, was largely colonized by people from Mo in northern Helgeland, and they have, to a greater extent than others, used roots for fodder, in particular of various kinds of ferns (*blom*, which they called *jeiske*), more so than others here, which may have used roots [only] in times of need, if they were accessible.” (NEG 11: 1820). The tradition’s concentration to the interior valley area is confirmed by a second record: “(…) but the old woman I mentioned above tells me than in the old days, fern roots were used for fodder (in Beiarn), but she could not provide further information.” (NFA O.A. Høeg 16, 1938). A later comment from the same informant was obviously based on a new source, noting that rhizomes “was used for cows. The roots were used raw, but first they were crushed” (…) “It was an important source of additional fodder in the upper part of Beiarn.” (NFS O.A. Høeg 16, 1948; [4]: 326); “The *mollfór-*roots were chopped, mixed with other fodder, and warm water was poured over to soak the mixture. All kinds were collected, usually in spring, and used little by little. It was rescue fodder.” ([4]: 326).

In the NEG material, a man from Saltdal answered that fern rhizomes had not been used in his area, but he knew the practice from Vatnbygda in neighbouring Fauske, where it had “in the past been used every year, and in substantial quantity. It was considered a good fodder.” (NEG 11: 2810). Note, however, that rhizome use was recorded from Saltdal by Axel Hagemann in the late 19th century ([53]: 44).

Three records from Bodø area provide only snippets of information: “*Mollfór* was collected as an additional fodder.” ([4]: 326); and at Kjerringøy: “Old people say that *moltfór* was used for cattle.” ([4]: 327); “In the war years of 1914–18 some people would go to the “*moltfórskogen*” [‘the fern rhizome forest’] and fetch roots for cattle fodder.” ([4]: 327).

A 1950’s record from Sørfold refers to use in the late 19th century: “A woman who is now in her eighties said that in her childhood in Sørfold it was common to collect [fern] roots to feed livestock. They held these roots to be a very fine fodder, equal to hay. Now the use has ceased. *Moldfóret* was stored in sheds.” ([4]: 327). A reply in the NEG material extends the last use in Sørfold well into the 20th century: “During the previous world war in 1914–1918, when I lived in Sagfjordgrenda in Sørfolla, I participated in harvesting ferns for fodder. We scythed the grass [fronds] and dried it as other grass, or rather somewhat less so it should not fall apart, and stored it in the shed just as ordinary hay. I did not collect fern roots (*moldfor*) but some of my neighbours did.” (NEG 11: 1933). Yet another source from this area shows that at least two fern species were used as sources of fern rhizomes for fodder: “Where there were slopes with ferns or blom, they dug up the roots, which were called *telli* or *moltfôr*, according to which species were used. *Moltfôr* in particular was considered a good supplementary fodder, but mostly, it had to be carried or dragged a rather long way.” ([71]: 82). In this area, *telli* is likely to refer to *Matteuccia struthiopteris* (cf. [54]): *moltfôr* may refer to a *Dryopteris* species.

In Steigen, ferns constituted part of the fodder available. According to a note made in the 1950’s, the fodder for “two or three cows and some minor livestock consisted largely of grass and *blom* [ferns] collected in the outfield areas, and a little *molfôr* [fern rhizomes]” ([77]: 100). A record in the NEG material confirms that rhizomes were well known as fodder: “Here in our area, the roots of *blomgresset* [fern fronds] have been used since time immemorial as a kind of additional fodder.” (NEG 11: 1644).

The use of *Dryopteris expansa* as *moldfôr* in Hamarøy has been mentioned above. A second record from this area provides little in terms of details, saying only that “The root of a fern species was dug up in autumn, for fodder.” ([4]: 327). Tradition in neighbouring Tysfjord approaches that found further north, noting that a poisonous kind had to be avoided: “The roots of *storblomen* [large fern, probably *Matteuccia struthiopteris*], which can grow as tall as a man, was the finest livestock fodder. They were dug up in summer, placed to dry below an overhanging cliff, pulled home in winter, and used as fodder during the spring shortage. One kind was called *blindmoltfór* and was poisonous, the animals got blind from eating it.” ([4]: 327).

In Tjeldsund, rhizomes were only resorted to in lean years: “Roots have never been collected in any quantity in this area as far as people can remember. Some *moldfor* was collected during the period of spring shortage, but now this has completely ceased.” (NEG 11: 1796). The sole record from Lødingen is even less detailed:, noting only that “*Mollfor*, *Blomrot*. The root is used as additional fodder.” (NFS O.A. Høeg 485; 1938; cf. [4]: 327).

Similar use prevailed in the Lofoten islands, e.g. in Vågan: “Of roots [as fodder], I have only heard about *blomrøtter* [fern rhizomes].” (…) “The subterranean stem was as thick as the handled.” (NEG 11: 2463). “The roots of *blomm*” (…) “as *mollfór*, preferably for cows. It was usually chopped, and warm water poured over (to soften).” ([4]: 326); “*Mollfór* is collected in a sack during the autumn, and used in spring. If the roots are somewhat flattened, they are called *blindmollfór*, because pigs and sheep will become blind if they eat it.” ([4]: 326); “*Moldfór*. It was the white roots which were used to feed livestock. Some ferns have dark roots. They are called *blindmoldfór* and were not used.” ([4]: 326–327); “They discriminated between *mollfór* and *blindmollfór*, mainly based on the colour. The *mollfór* was softened, i.e. boiled together with fish remains etc.” ([4]: 327).

Tradition at Sortland in Vesterålen was much the same, adding a third kind (*pilmollfór*), which is likely to have been *Matteuccia struthiopteris*: “*Mollfór* is the root of ferns, which is used as fodder for cows and sheep. *Pilmollfór* is less good, it grew among the stones in damp areas. *Blindmollfór*, it caused the cattle to go blind.” ([4]: 327). A more recent record from the same area states that people “would also fetch something from the slopes which was called *moldfôr*, it is a root that stands under the ferns (*blomen*) you will often find there.” ([78]: 124). Ingvald Johansen provides similar information from this area, noting that “The old ones discriminated between three kinds of *moldfôr*. First it was *godmoldfôr* [‘good *moldfôr*’], which they were primarily harvesting. Then there was *blindmoldfôr* [‘blind *moldfôr*’], which was confusingly similar, though there was a difference in the leaves [fronds]. The old folks believed that the animals would become blind from eating it. A third kind was *pil* [‘arrow’]. Of this kind, there was a fair quantity on the slope above the uppermost farms in the Holmstaddalen valley.” (…) «This kind was called *pil* because when the fern had withered, some high leaves were left standing, which looked like the tail of a bird.” ([32]: 100–101). The latter characteristic leaves no doubt that *pil* was *Matteuccia struthiopteris*, where the deviant, stiff and brown, fertile leaves remain standing into autumn and winter. Some additional information is found in my own material, again noting that people discriminated between different kinds of *moldfôr*. “My father could tell the plants apart, on the *blom* [the fronds]. I remember he told me, the leaves were different.» (…) «The one we used, was *godmollfor*.” It was last used in 1945 or thereabout: “He was here just after the war and collected some *mollfor*. Since then, I have not heard about anyone who did so.» (EBATA 2009:4).

At Hadsel in Vesterålen, rhizomes were commonly used: “I happened to meet two elder females in the forest picking berries, and used the opportunity to ask them about the use of fern rhizomes in the past (they are not collected by anyone nowadays). I was told that it was *ormetelgen* [= *Dryopteris filix-mas*] they used and designated as *moldfór. Skogburkne* [= *Athyrium filix-femina*] and *geittelg* [= *Dryopteris dilatata*] they called *blindmoldfór*. I suppose it is *geittelg* that elsewhere in Hadsel are called *pil* and *blindpil* due to the shape of the leaves.” ([4]: 327). The identification of *blindpil* as partially referring to *Dryopteris dilata* has no support elsewhere, and is probably a misunderstanding; as is the explanation; the fronds bear no resemblance to an arrow (*pil*). In addition, the plants found in this area are now regarded as belonging *D. expansa* rather than *D. dilata*, and as shown by records elsewhere, there is nothing to suggest that the latter species was harmful. People may, however, have found that it looked confusingly similar to *Athyrium filix-femina*. Another account from this area, cited above, is detailed in terms of the utensils used, and less so in terms of the result: “The roots are cut away from the plant [rhizome]. They stored it in large piles until it was transported home.” ([4]: 327).

Traditions further west, in Bø, were rather similar: “*Mollfór* was also collected during the war 1940–1945. They had *mollfór* and *blindmollfór*.” ([4]: 326). In the NEG material, the question of what kinds of roots were used for fodder received an extensive answer: “Of roots, people here have collected fern roots (*blom*). It is not used now, and not during the last world war.” (…) “I am not sure when the practice ended, but it has been used during the last 70 years. The roots of ferns are called *moldfor*. There were some roots that were called *blindfor*, and which are poisonous for animals. It looks sharp [probably referring to the spiny protuberances at the basal part of the stipes of *Athyrium filix-femina*]. The real [i.e. useful] ones are round, long roots which are green, and have a sweet taste. I do not know which fern species these roots belonged to.” (NEG 11: 2486, partly cited in [4]: 326). Two further records from Bø merely confirm that rhizomes were collected, noting “fern roots” among additional fodders used (NFS O.A. Høeg 496; 1946) and that “previously, it was common practice to collect” (…) “fern roots” (NFS O.A. Høeg 497; 1946).

Two records from Øksnes confirm this pattern: At Dyrøya, people “also collected moldfor” as additional fodder (EBABM 1990:9). A slightly more detailed record is available from Langøya: “From *storblomen* [‘the large fern’] they dug *moldrot* for the sheep; it was so sweet that the children would chew it.” (EBABM 1990:9, [63]: 14). A third record from the same area added that people had to be careful to avoid a harmful species: «Before his time, people collected special plants in the outfield areas, *mollfor*, [from] the fern which is called *blom* here. [One] has to know this *blom*, because there was one [kind] they called *blindmollfor*. If it was used, the animals could become blind for shorter or longer periods of time.” The informant did not know what species this was, but records from other areas point strongly to *Athyrium filix-femina* (EBABM 1990:10; [63]: 14).

#### Troms

In Harstad, the use of fern rhizomes continued until about 1910, but: “It was very little used. I can only remember a single occasion when my father and I went [to collect it].” “We went to the mountain slope to take up *mollfor*.” ([5]: 378). Commenting on this, my grandma added: “It was something they dug up and boiled in the large kettle in the barn, they talked about *mollfor*. But I do not really know what it was.” ([5]: 378). A record from the adjacent mainland, in Skånland, has been partly cited above; in this case, rhizomes “were also used during World War II” ([4]: 328).

At Ibestad, people discriminated between three kinds of ferns when harvesting rhizomes for fodder, according to a 1950’s record: “The old people had three names for the *blom* [ferns] that were collected for fodder, namely *moldfor*, *telg* and *teisk*. But I do not know what ferns the names refer to.” (…). “*Moldfor* was considered a fine fodder, but *telg* and *teisk* were less good” (NEG 11:3413). Unfortunately, the record provides no clues as to the identity of the three (or possibly more) species collected. In this area, the use of fern rhizomes may have ceased in the first decades of the 20th century: “In the old days, *mollfór* was considered an additional fodder, not a rescue fodder, for at some farms, it was gathered every year. But nothing has been collected during the last 40 years.” (NEG 11: 3413). In Gratangen, a toponym reflects the tradition of collecting fern rhizomes: “There, you will also find *Moldfôrholla*, where they dug roots, for fodder.” (EBATA 2005:9). A record from Salangen has been cited above.

Fern rhizomes were also collected at Espenesbogen in Dyrøy, but under a deviant name – which perhaps refer to the plants as such: “*Telgen*, its nutrient store [i.e., the rhizomes] was used as cattle fodder during the period of spring shortage. We had a place-name *Telgelia*.” (EBATA 2014:27). Unidentified fern rhizomes were also collected at the major island of Senja: “(…) but in Vesterålen and in Berg, during the spring shortage, they dug up the roots of the large fern” (…) “in the slopes, for fodder. This they called *moldfór*.” (NFS O.A. Høeg 757; 1961, partly cited in [4]: 328). At the southern part of Senja (in Tranøy), people utilized both fronds and rhizomes as fodder: “*Blom* (fern) was harvested, dried and used as livestock fodder. In addition, the roots were dug up for fodder (*moldfor*), and these practices may have been a good reason for making toponyms.” (…) «In one locality [out of six listed], ferns are absent today, but one informant (born 1906) knew that they had dug *moldfôr* here in the time of his parents” ([79]: 187).

In Bardu, two kinds of rhizomes were recognized: “Two kinds of *blom* [ferns], one was *mollforrot*, the other was *storblom* [large fern]” (…) “It was used before her time. They cleaned the root [rhizome] and cleaved it in two halves.” Inferring that *mollforrot* came from a smaller fern, the sources of rhizomes for fodder may have been *Dryopteris expansa* or *D. filix-mas*. In Salangsdalen, such use was rare, at least in the 20th century: “[We did not use] *blomrot* in any quantity, only now and then.” (EBABM 1989:11). The larger species may also have found local use: “*Strutsevinge* [= *Matteuccia struthiopteris*] we call *blomrot*.” (…) “It was used to somewhat after 1900.” ([4]: 328). A more extensive record confirms this, adding two further species, of which only one was used fodder: “*Teljerot* (*telg*, also called *storblomrot*, which must be *strussveng* [= *Matteuccia struthiopteris*], has a very big root, almost like a human head, and the *blom* [frond] is high and large, the largest of all kinds of *blom*. Previously, the root was used for fodder. In this case, *ormetelg* [= *Dryopteris filix-mas*] was also used: “*Moldfórrota*” (…) “has a smaller root, and the *blom* [frond] is also smaller. The lowermost part of the stem is smooth, without spines. It is the best root for livestock fodder, but it is less frequent in the forest.” As elsewhere, *Athyrium filix-femina* was known, but not used: “*Teskrota*” (…) “deviates from the others in that root neck, the lowermost part of the stem, is flatter and has spines along the rim. It is of about the same size as the previous one, both in terms of root and leaf. It forms bundles in the forest. The root was not used for fodder.” ([4]: 328). Unlike *Dryopteris* spp. and *Matteuccia struthiopteris*, *Athyrium filix-femina* rhizomes are often densely spaced, thus forming “bundles”.

At Rostadalen in Målselv, people “Took up roots [rhizomes] as additional fodder. *Mollfor*. [It was] cleaned and chopped into pieces.” (EBABM 1989:2; [63]: 14). A record from nearby Dividalen is more detailed: “*Blomrot* was used for livestock. There are two kinds of root, *mollforrot* and *blomrot. Mollforrota* was smaller. More gracile and with paler leaves.” (EBABM 1989:5–6; [63]: 14). The former may have been *Dryopteris expansa*, with its pale green fronds. A second record from Målselv specified that one kind was harmful: “*Mollfórrot*, that is the root of *ormegras*, was used as a rescue fodder. One kind was considered to be poisonous.” ([4]: 328).

People in Balsfjord also discriminated between several kinds of fern rhizomes (cf. above): “The *mållfor-*root was a fine fodder during the spring shortage. *Telg* and *tisk* are useless.” (…) “The base of the leaf stems are round in *mållfór*, whereas it is triangular in *telg* and *tisk*, somewhat broader in *telg*.” (NFS O.A. Høeg 101; 1938; [4]: 328). People living at the interior part of Balsfjord could row to Nordkjosbotn to collect fern rhizomes (NEG 11: 20628), presumably from the large stands of *Matteuccia struthiopteris* along the Nordkjoselva river.

In Tromsø, fern rhizomes are included in a list of various additional fodders used by tenants at Oldervik in the 18th century: “So there they have utizilized *skav* [rowan bark] and *Multefoder* [fern rhizomes] for their livestock, (…)” ([80]: 7). A record from Kvaløya provides little in terms of details: “*Mollfór* is t*elg*, the root [of ferns].” ([4]: 328).

At Kåfjord, the use of fern rhizomes had ceased long before the NEG questionnaire was distributed in 1948. “In the valleys of Kåfjorddalen and Manndalen, roots have been little used recently. Only way back in time, fern rhizomes were used in quantity. Younger people had no idea when I asked them. But older people could remember it, and according to their account, the practice was as in Nordreisa.” (NEG 11: 16673). An unusual array of records is available for the latter area. Excerpts of a detailed account given by Yngvar Mejland have been cited above. According to him, fern rhizomes “were regarded as a good fodder, and previously, until about 1915, it was used as an additional fodder at most farms in the area.” The species harvested were *Dryopteris filix-mas* and *Matteuccia struthiopteris*. “Only these two kinds of fern are used. At least until 1935, *mollfór* was a frequently used emergency fodder. It has been less used since then, but due to the lack of manpower, people will not go far to collect it. Previously (until about 1935) people could travel tens of km to “cut *mollfór*”” (NEG 11: 4688).

A man from the Nordreisa valley of had participated in collecting fern rhizomes in the early 1940’s. Asked if he knew the tradition of using *moldfôr*, he provided a brief account: “Oh yes. In autumn, we prepared large piles of *mollfor* in the forest, which we brought home [in winter]. And we continued with this until 1940.» (…) «I accompanied my father-in-law collecting *mollfor*. It was mid-summer’s eve, and the snow was still piled so deep it reached the belly of the horse.” (…) “We pulled up *mollfor* in autumn and stacked it, and brought it home in spring or whenever it suited us. We could also fetch it earlier in winter; there was no fixed date for doing so. It was when it was needed. In Finnish, *mollfor* is called *gaiski*.” He went on to provide further details, noting that there were different kinds: “There is *sau-mollfor* [‘sheep-mollfor’] and *ku-mollfor* [‘cow-mollfor]. *Sau-mollfor* is a different kind. It is somewhat smaller, but it is much better. *Ku-mollfor* is more dry, and it is much larger. I can show you the two kinds in summer” [which, unfortunately, seemingly was not done] (EBABM 1991:3; [63]: 14). Still, his description of the former may suggest *Dryopteris expansa* or *D. filix-mas*, favourite sources of *moldfôr* in other areas, whereas the latter, large kind may have been *Matteuccia struthiopteris*, which is abundantly available in the area. A woman from the same area, but at a farm closer to the sea, seemingly had less knowledge of such use, and perhaps the locals did not know which species to collect – at the coast, other kinds of fodder (e.g. kelp) are easily available: “I remember once they collected *mollfor* beneath the Solheim-bakken slope, it was chopped and made [ready]. But the cows would not eat it; they were not that hungry.” (EBABM 1991:2). At Kvænangen, in the far northeast, Troms, fern rhizomes were previously an important source of fodder. “Now, all use of roots as fodder has ceased in Kvænangen. The practice was abandoned before the last war.” (NEG 11: 16672).

#### Finnmark

Only a handful of records provide information on the use of fern rhizomes in the far north of Norway. Some notes on the use of fern rhizomes is found in Fredrik Rode’s 1832 account of the agriculture in Finnmark, the northernmost county of Norway [81], repeated in his 1842 general account of the county [64], but in both cases referring mostly to the Alta area: “Another resource, which also is available only to those who live in areas with fertile soils and forest, is the so-called *Molfoder* (properly *Muldfoder*, from *Muld*, soil), which is the root of a plant, that look like the well-known *Ormegræs*. A variety of it is called *Groste* and is considered inferior.” ([64]: 140).

At Bognelvdalen in Alta, rhizomes were a well-known source of fodder in times of need. Asked if (birch) leaves were used, my female informant replied: “No, not leaves. If it was a bad year, we had a place where they dug up something they called *grofte* and *mollfor*. And the cows were very fond of it.” She was not sure if these terms designated two different species; *grofte* may have been used to designate the plant as such: “No, (…) it was two different things. It was the root. It [the fodder] was *mollfor*, and it was like a small kohlrabi.” They had to cross the Bognelva river to find it, which means that in this case, *Matteuccia struthiopteris* can be ruled out – it is abundant on the valley floor on both sides of the river. She could not offer any other details than that the species used was “stiff”, which may point to *Dryopteris filix-mas*, with its rather dry and hard fronds. Perhaps other species were known or used as well, as suggested by an additional comment: “There were several kinds. One was more sharp, and a little darker” – which partly complies with the descriptions given elsewhere of *Athyrium filix-femina* and the “spines” at the base of its stipes. The last use of fern rhizomes had occurred just after World War II (EBATA 2007:45).

The NEG material includes a record from Eibydalen in central Alta: “One has collected some fern roots for fodder.” (…) “The best kind was *ormegresset*, but one would also collect som *muldfor. Trollmollfor* had to be avoided. One recognized it by the spines at the base of the leaves» (NEG 11: 21066) – a description that once again identifies *Athyrium filix-femina* as the harmful species. At Kviby, the larger ferns were infrequent, but the locals “knew that about 100 years ago, people had collected some fern roots in a slope” (NEG 11: 22284).

At Nuvsvåg in Loppa, “the larger ferns do not grow here in any quantity, so their roots were but little used.” (NEG 11: 21367), whereas an informant from Bergsfjord had no knowledge of such use (NEG 11: 9794). A sparse or absent tradition of using fern rhizomes for fodder in Loppa is confirmed by an 18th century source. According to a letter from vicar S. Bang to bishop J.E. Gunnerus, dated July 20, 1763, “moldfor is not used in Loppa” ([82]: 29).

Further east, at least people in Lebesby collected fern rhizomes for fodder, under a deviant name, *blommerøtter* (NFS OAH 691, 1955; [4]: 329). An informant from Tana has been quoted above. Two further informants from this area, of Finnish ethnic origin, provided nothing in terms of identification: “Some fern roots – *kaiski* in Finnish – have been used, but just a little” (NEG 11: 19898). At Sør-Varanger in the far east of Finnmark, two informants from Neiden and Jarfjord both both noted that the larger ferns were infrequent, and thus they had no knowledge of using the rhizomes for fodder (NEG 11: 21187, 21532).

### Fern species avoided during rhizome collection

Rhizomes of some fern species are poisonous or otherwise harmful if used as fodder (Table 5). According to Jens Holmboe [3], this was common knowledge among the farmers he interviewed during his study of fern rhizomes and their use.

In North Norway, such species were sometimes given separate names, *trollrot* (“troll root”) and *blindrot* (“blind root”) are typical examples ([3]: 765–766). Such names usually refer to *Athyrium filix-femina*. According to farmers in both Nordland and Troms, such rhizomes would cause blindness in animals if they were used as fodder. Rhizomes of *A. filix-femina* were recognized by the hard, spiny protuberances or “teeth” of the basal stipes. In Nordreisa in Troms, North Norway, the “useless” large species was designated as *trollmoldfôr* (‘troll soil fodder’), to separate it from the useful species, i.e. *Dryopteris* and *Matteuccia struthiopteris* ([19]: 219). In other areas, *moldfôr* was used as a general term for fern rhizomes (e.g. [5]: 378), but some kinds were used and others not.

Some records merely show that people knew or believed that some kinds of *moldfôr* were harmful, e.g. at Bleik in Andøy, Nordland: “Grandma Anna tolds us about *mollfor* which was poisonous, and she called it *blindfor*. The animals could loose their sight.” (EBATA 2005:51).

### Ferns and fern rhizomes in the tradition of the Sámi and Finnish ethnic minorities

Within Norway, the use of fern rhizomes for fodder is first and foremost a tradition found among the ethnic Norwegians. The Sámi were much later to take up agriculture, learning the techniques from their Norwegian (and sometimes Finnish) neighbours, including the use of fern rhizomes as an additional source of fodder. Most, but not all Sámi fern names are adaptions of Norwegian terms. In all likelihood, the species selected for fodder use were also the same, although the available material is fragmentary and partly misleading. In the far north (Troms and Finnmark), ferns and fern rhizomes are mostly designated as *gáiski*, translated as “the root [rhizome] of various fern species” by Just Qvigstad ([34]: 320). From areas further south, in Nordland and Troms, he noted two deviant terms for fern rhizomes, in present-day spelling: *rehppe* and *delgi* ([34]: 318, 329, [35]; 41, 125).

In his Sámi dictionary, Jens Friis [83] identified the source species as *Pteridium aquilinum*, which is obviously wrong, since the species is absent from all the major Sámi settlement areas. It is only known from a few stations within the two northernmost counties of Norway – both in Troms, and none at all in Finnmark. In his *Flora norvegica*, Johan Ernst Gunnerus [43, 44] mentioned a couple of Sámi *gáiski* terms for ferns and fern rhizomes, specified (in obsolete spelling) as *gaiske banekætta* (‘toothless fern rhizome’) and *sapak gaiske* (‘black fern rhizome’), the latter combining the adjective *sáhppat*, which is more correctly translated as dark or blackish blue, the kind of colour seen in some ripening berries, or in effusions of blood, and *gáiski*, ‘fern (rhizome)’. Both were identified as *Matteuccia struthiopteris*, whereas *Athyrium filix-femina* was called *stálogáiski* and *bánegáiski*. The former is pejorative, *stállu* being an evil but stupid giant that troubled the Sámi. The latter refers to a characteristic of the rhizomes – they have “spines” or “teeth”, cf. the Sámi term *bátni*, ‘tooth’. As we have seen, the absence of “teeth” on the rhizomes of *Matteuccia* and other useful fern species is confirmed by Holmboe’s study of how Norwegian farmers discerned between the useful and harmful kinds ([3], and numerous later records. Vernacular names may also refer to ecology, e.g. *juovvagáiski* (‘scree fern’) in Kåfjord, Troms, supposedly for *Dryopteris filix-mas* (EBATA 2003:3).

A record form Tysfjord refers primarily to Sámi use of fern rhizomes, but offers nothing in terms of identification of the species used: “Especially by the Lapps [Sámi] quite a lot of *moltfór* is collected in spring and autumn, but mostly in spring. It is taken from *småblomme*, and this seems to refer to all ferns except (…)” *Pteridium aquilinum* ([4]: 327).

Locally, people discerned between several different kinds of *gáiski*, mostly unidentified in the sources, though some names occur. Knud Leem suggested that *gajsske* (in his spelling) referred to *Dryopteris filix-mas*, with no geographical location given ([84]: 319), and the identification seems likely, since the rhizomes are frequently used in Norwegian tradition. In Kvænangen, Troms, people recognized at least three different kinds, referred to, in Qvigstad’s spelling, as *savcagaiske* (‘sheep fern rhizome’), *gusagaiske* (‘cow fern rhizomes’), and *truollagaiske*, i.e. *ruollagáiski* (‘troll fern rhizome’), the latter identified as *Athyrium filix-femina*, with the pejorative *(t)ruolla-* suggesting that is was considered harmful – as it is in Norwegian tradition [34].

Within Norway, Finnish traditions related to ferns and fern rhizomes are closely related to that of their Norwegian neighbours, and probably largely adopted from them. The predominant name used for ferns is *kaiski*, which was used e.g. in Nordreisa Troms (EBABM 1991:3), and in Alta, Porsanger and Tana (NEG 11: 19898), Finnmark. It is also found in some toponyms, e.g. Kaiskikuru (‘fern valley’) in Nordreisa ([85]: 66) and the somewhat abridged Kaiskuru in Alta, and Kaiskiruto (‘the fern thicket’) in Porsanger, Finnmark ([85]: 194, [86]: 11). In Finland, *kaiska* has been used as a vernacular name for *Gymnocarpium dryopteris* and *Matteuccia struthiopteris* [87] – the latter one of the main sources of fern rhizomes for fodder in northern Norway.

### Ecological consequences

Large-scale extraction of fern rhizomes could deplete stands. Locally, people referred to past use of *moldfôr*, noting that rhizomes were no longer available, e.g. at Hillesøy in Tromsø: “Here, there is but little of the larger ferns, and they were eradicated at an early date. People knew than *blom* had previously been used.” (NEG 11: 22651). Similar information was offered at Lenangen, also in Tromsø: “In the past, one used *blom* here. It was so heavily exploited that it no longer pays the effort to collect it.” (NEG 11: 22708). The situation in Kvænangen, in northermost Troms, may have been the same, for “Some said that people may have used fern rhizomes some time before the war, but the ferns were eradicated, so it did not pay to search for them.” (NEG 11: 16672).

Yngvar Mejland recorded the botanical consequences of extensive harvesting of fern rhizomes in Nordreisa: “Along the sea, and in the lower part of the valley, the stands have been so heavily utilized that *ormetelgen* [= *Dryopteris filix-mas*] is rare here, whereas *strutsevingen* [= *Matteuccia struthiopteris*] is [still] found in large stands.” (NEG 11: 4688). Thus, the extensive past use of fern rhizomes may have had consequences in terms of species composition in the vegetation. Jens Holmboe ([3]: 767) noted that *Athyrium filix-femina* could predominate over large areas, possibly due to the selective collection of other large fern species, leaving it untouched and thriving.

## Discussion

As shown above, the majority of sources referring to the use of fern rhizomes as fodder provide little or nothing in terms of identifying the species used. Only botanists have ventured to do this. With few exceptions, they identify *Dryopteris filix-mas* and *Matteuccia struthiopteris* as the main source plants. The latter may form vast large stands in the larger river valleys, and is thus easily available for exploitation. *Dryopteris filix-mas* is usually found growing among other ferns, and requires more effort in terms of locating. It is more frequent in the interior valleys than at the coast, and both the study of Jens Holmboe [3] and Mejland’s records (in the NEG archive) derive mostly from the former area. At the coast, *Dryopteris expansa* is a much more frequent species, and as shown by some recent records, it was locally the preferred source of fern rhizomes. Thus, for northern Norway in general, the data so far available may have over-estimated the use of *D. filix-mas,* and overlooked *D. expansa* as an important, alternative source. Rhizomes of *Dryopteris filix-mas* and *Matteuccia struthiopteris* were used for fodder in Sweden as well, as recorded by Anders Jahan Retzius in 1806 ([88]: 481, 540), and Ernst Henning in 1889 ([89]: 24), but were probably of restricted importance, forming parallels to the scattered and infrequent use of fern rhizomes in southern Norway. Contrary to this, northern Norway, and in particular the counties of Nordland and Troms, stand out as the core area of thizome use, in accordance with the long winters encountered in the north, and a humid climate supporting large stands of ferns. This is also the only area where the use of fern rhizomes survived into the second half of the 20th century.

Although folk tradition in Norway singles out *Athyrium filix-femina* as harmful and poisonous, allegedly causing lame hind legs and blindness in livestock, there is hardly any toxicological data to support this in terms of the rhizomes. Fresh shoots are poisonous due to their contents of thiaminase, which is destroyed if dried or boiled – which the rhizomes usually were, before being served as fodder. Feeding livestock with fresh rhizomes collected in late spring, complete with their sprouting buds or fiddleheads, could perhaps lead to poisoning. *Matteuccia struthiopteris* is one of few fern species gathered in any quantity (as young shoots or “fiddleheads”) as food for humans, e,g in North America ([90]: 1773), and in Japan. To some extent, *Athyrium filix-femina* is used in the same way the U.S. *Diplazium esculentum* (Retz.) Sw. (syn. *Athyrium esculentum*) is eaten in East Asia and the Pacific area. As *pohole*, it is a food speciality of Hawaii ([90]: 1837). Such use is hardly compatible with serious poisoning, unless the metabolic pathways in humans and livestock differ considerably, as they do for instance for coumarin in grasses and other plants (14], [91]: 444). In fact, at least the blindness attributed to *Athyrium filix-femina* in Norway may as well be due to using *Dryopteris filix-mas* or other *Dryopteris* species as fooder ([92, 93]: 1247–1248). *D. filix-mas* is known to be toxic, as also suggested by its well-known antihelmintic properties, and ingestion of the apical buds of the rhizomes may cause severe disturbance of the digestive system, blindness, and death [94, 95].

The past use of fern rhizomes for fodder is difficult to quantify. Based on data collected for taxation purposes in the 1860’s, I have previously extracted information on the use of various additional fodders in the municipalities of Harstad and Kvæfjord in southwest Troms, North Norway [96]. According to this data set, 48 of 248 farms included were listed as having access to fern rhizomes, much fewer than those which had access to kelp or leaves, bark and twigs from deciduous trees (e.g. *Betula pubescens* Ehrh., *Sorbus aucuparia* L.). On the other hand, fern rhizomes were eagerly collected when available – 94 % of the 48 farms with access to fern rhizomes reported that this resource was “utilized in full” [96].

The extensive Norwegian use of fern rhizomes for fodder seemingly has no parallel anywhere else. There is no information on such use in any of the major scientific databases (e.g. Scopus, Web of scienc). Although such use has been recorded in Sweden and Finland as well, it was of little importance there, and rarely practiced. It was hardly practiced in the Norse overseas settlements eiher – for the simple reason that neither the Orkney or Shetland islands, the Faroes, Iceland or Greenland provide much in terms of ferns for harvesting. The suitable species are, as pointed out already by Fredrik Rode in 1832, in his case referring to Finnmark, mainly found in forested areas, and for this reason, to quote Rode, it was “an additional fodder which is only available to people living in areas, where woodlands are found” ([81]: 26). Ferns have found use for various purposes worldwide, e.g. for food and in herbal medicine in North America [97]. Contrary to this, neither immigrants nor the indigenous groups of North America had any need for fern rhizomes for fodder. In northern Eurasia, such use seems to have been rare or absent outside Norway.

## Conclusion

Fern rhizomes have a long tradition as one of many kinds of additional fodders in Norway, mainly among the ethnic Norwegians. Preferred source species were *Matteuccia struthiopteris* and *Dryopteris filix-mas*, more locally also *Dryopteris expansa*, whereas *Athyrium filix-femina* was generally, but not always, considered poisonous and harmful, and thus avoided. The core area of such use is found in the two North Norwegian counties of Nordland and Troms, where the practice locally survived into the 1950’s.

Fern rhizomes are only likely to find fodder use in northern areas combining at least some agriculture with long winters and woodlands supporting large stands of ferns – and Norway, in particular its northern parts, fits this description perfectly. There are hardly any other areas anywhere where agriculture is carried out at such high latitudes, and still within birch-dominated forest areas providing access to abundant stands of some of the larger fern species, and thus their rhizomes.
